# A natural history and copula-based joint model for regional and distant breast cancer metastasis

**DOI:** 10.1177/09622802221122410

**Published:** 2022-09-18

**Authors:** Alessandro Gasparini, Keith Humphreys

**Affiliations:** Department of Medical Epidemiology and Biostatistics, 27106 Karolinska Institutet, Stockholm, Sweden

**Keywords:** Natural history model, copula, breast cancer, survival analysis, microsimulation

## Abstract

The few existing statistical models of breast cancer recurrence and progression to distant metastasis are predominantly based on multi-state modelling. While useful for summarising the risk of recurrence, these provide limited insight into the underlying biological mechanisms and have limited use for understanding the implications of population-level interventions. We develop an alternative, novel, and parsimonious approach for modelling latent tumour growth and spread to local and distant metastasis, based on a natural history model with biologically inspired components. We include marginal sub-models for local and distant breast cancer metastasis, jointly modelled using a copula function. Different formulations (and correlation shapes) are allowed, thus we can incorporate and directly model the correlation between local and distant metastasis flexibly and efficiently. Submodels for the latent cancer growth, the detection process, and screening sensitivity, together with random effects to account for between-patients heterogeneity, are included. Although relying on several parametric assumptions, the joint copula model can be useful for understanding – potentially latent – disease dynamics, obtaining patient-specific, model-based predictions, and studying interventions at a population level, for example, using microsimulation. We illustrate this approach using data from a Swedish population-based case-control study of postmenopausal breast cancer, including examples of useful model-based predictions.

## 1 Introduction

The clinical staging of cancer is strongly correlated with prognosis and contributes to the choice of specific treatment regimes. In the United States, for breast cancers diagnosed between 2010 and 2016, the 5-year relative survival rates were 99%, 86% and 28% for localised, regional and distant stages, respectively (figures from www.cancer.org). Similarly, in Sweden, Bower et al.^1^ reported relative survival rates of 97–98%, 92–94%, 75–79% and 25–33% for women diagnosed with Stage I to IV breast cancer, based on data between 1992 and 2012 and obtained from the Breast Cancer Data Base Sweden (BCBaSe). Few breast cancers are fortunately diagnosed with distant spread, but for those without distant spread at diagnosis, progression to distant metastatic cancer can occur years after diagnosis of the primary tumour, and unfortunately, once being diagnosed with distant metastases, prognosis is still, these days, poor.^2^ Different subtypes of cancer are associated with different patterns of recurrence (with distant metastases).^3^ Estrogen receptor (ER) negative cancers are associated with early recurrence, whilst ER-positive cancers are associated with later recurrence, but with more than 5 years sustained risk.^4^ A deep understanding of metastatic recurrence is important for improving the prevention and treatment of breast cancer.

There are though few statistical models of breast cancer recurrence or progression to distant metastasis. Most of these are based on multi-state modelling.^5,6^ Whilst these approaches are very useful for summarising the risk of recurrence, they do not provide insights into the underlying biological mechanisms and have limited use in terms of understanding the implications of interventions (e.g. modifications to screening). In this paper, we describe an alternative approach to modelling (lymph node and distant) metastases, based on a biologically-inspired continuous tumour growth model. The strength of continuous growth models lies in their flexibility and parsimony. Although they rely on strong parametric assumptions they have the potential to be useful for understanding disease dynamics.^7–15^ Early continuous growth models, applied to breast cancer screening data, combined a tumour growth model with a continuous function of screening sensitivity and accommodated random effects to account for heterogeneity in tumour growth. Isheden et al. described a joint model of tumour size and lymph node spread, and Gasparini and Humphreys developed a model of tumour size and distant metastatic spread.^14,15^ To the best of our knowledge, no natural history model that jointly accommodates both local and distant metastatic spread alongside latent tumour growth has been proposed in the literature so far. Specifically, the model introduced in this paper is developed for breast cancer incident cases collected in a population of women in which screening is offered.

First, we describe the model for tumour growth in Section 2. We then describe the models for spread to the lymph nodes and distant metastatic spread in Sections 3 and 4, respectively; these models are based on previous work.^14,15^ We bring the models together using a copula-based approach (Section 5), and then formulate likelihood functions, first in the absence of screening (Section 6), and then for a screened population (Section 7). Possible choices for the copula function are discussed in Section 8. In Section 9, we summarise Monte Carlo simulation studies which check the computing algorithms. Results and additional details of the simulation studies are provided in the Supplementary Material. Model-based predictions that can be obtained after fitting the joint model are introduced and described in Section 10. In Section 11, we analyse data from a Swedish population-based case-control study of postmenopausal breast cancer. We conclude the article with a discussion in Section 12.

## 2 Modelling tumour growth

The growth of the primary tumour is assumed to follow an exponential function such that, for a tumour growing according to an inverse growth rate R=r, t years after onset it has volume $V(t|r)=V_{\text{Cell}}\exp\;(t/r)$, where $V_{\text{Cell}}$ represents an initial volume computed under the assumption that tumours are spherical and have a diameter $d_{\text{Cell}}$ = 0.01 mm.

Individual variation in growth rates is accounted for by assuming that inverse growth rates follow a Gamma distribution with shape $\tau_{1}$ and rate $\tau_{2}$:

(1) $$f_{R}(r)=\frac{\tau_{2}^{\tau_{1}}}{\Gamma (\tau_{1})}r^{\tau_{1}-1}\exp\;(-\tau_{2}r),\;r\geq 0,$$

where Γ(⋅) is the Gamma function.

We assume that tumours are detectable with non-zero probability from a given volume $V_{0}$ corresponding to a diameter $d_{0}$ = 0.5  mm. The process for detection (either via symptoms or screening) is described below and in Section 7. Given the exponential growth model described above, $t_{0}$ represents the time it takes for a single tumour cell to grow until it becomes detectable, where $V_{0}=V_{\text{Cell}}\exp\;(t_{0}/r)$.

As in previous work,^7,9,12,13^ we assume the following hazard function for time to symptomatic detection

(2) $$h_{T_{\text{det}}}(t)=\left\{ \begin{array}{cc} \eta V(t,r) & \text{if}\;t\geq t_{0}\\ 0 & \text{if}\;0\leq t<t_{0} \end{array}\right.$$

so that, after time $t_{0}$, the rate of symptomatic detection at time t is proportional to tumour volume, V(t,r), with proportionality constant η.

For an unscreened population, the density for tumour volume at symptomatic detection can, from the above assumptions, be calculated as follows:

(3) $$f_{V_{\text{det}}}(v)=\eta \tau_{1}\frac{\tau_{2}^{\tau_{1}}}{(\tau_{2}+\eta (v-V_{0}){)}^{\tau_{1}+1}},\;v>V_{0}$$

See Plevritis et al. for a derivation.^9^ Throughout the article, we use $V_{\text{det}}$ to denote a random variable representing tumour volume at symptomatic detection in the absence of screening.

## 3 Modelling spread to the lymph nodes

The model for spread to the lymph nodes (seeding) is based on a non-homogeneous Poisson Process with intensity function

(4) $$\lambda (t,r,s^{*})=s^{*}D(t,r{)}^{k_{N}}D^{\prime }(t,r),$$

where D(t,r) is the number of times the cells in the tumour have divided, $D^{\prime }(t,r)$ is the rate of cell division in the tumour and $s^{*}$ is a proportionality constant.^14^ The exponent $k_{N}$ (with $k_{N}\geq -1$) adds additional flexibility to the model, implying that metastatic spread can depend on higher powers of tumour mutation or that tumours mutate at an accelerating (or decelerating) rate; this phenomenon is referred to as *genomic instability*. In practice, we can either fix the value of $k_{N}$ or estimate it from data. Under the assumption of a time to clinical detectability of $t_{0}$, the corresponding cumulative intensity function for detectable lymph node metastases is

(5) $$\Lambda (t-t_{0},r,s)=s{\left[\log\;\left(\frac{V(t,r)}{V_{0}}\right)\right]}^{k_{N}+1},t\geq t_{0}$$

with $s=s^{*}/[(k_{N}+1)(\log\;2{)}^{k_{N}+1}]$.

Given that breast cancer is a heterogeneous disease, we assume that spread to the lymph nodes occurs at different rates for different women. Therefore, we assume that s follows a Gamma distribution with shape $\gamma_{1}$ and inverse scale $\gamma_{2}$ (using the same parameterisation as in equation (1)). Isheden et al.^14^ showed that the probability of N=n clinically detectable lymph nodes, given S=s, R=r, and a tumour volume V=v (at any time), is independent of both S and R and follows a negative binomial distribution NB(l,p) with size $l=\gamma_{1}$ and probability $p=1-[(\log\;(v/V_{0}{)}^{k_{N}+1}]/[(\log\;(v/V_{0}){)}^{k_{N}+1}+\gamma_{2}]$.

The probability of having N=n affected (and clinically detectable) lymph nodes given a tumour volume V=v is therefore calculated as

(6) $$P(N=n|V=v)=\frac{\Gamma (n+l)}{\Gamma (l)n!}p^{l}(1-p{)}^{n},$$

which can be used to calculate the probability of n detected lymph nodes given a tumour size at diagnosis.

## 4 Modelling distant metastatic spread

The model for time to distant metastatic spread is based on the same mathematical formulation as for lymph node spread; it is based on a non-homogeneous Poisson process, as in equation (4), but with parameters $\sigma^{*}$ and $k_{W}$ instead of $s^{*}$ and $k_{N}$. We however only use information from the first seeded/detected distant metastasis. In the absence of heterogeneity in the rate parameter, and under a number of assumptions about the time from distant metastatic seeding to detection of distant metastasis (assumed to be correlated to the growth rate of the primary tumour), for a fixed value of $\sigma =\sigma^{*}/[(k_{W}+1)(\log\;2{)}^{k_{W}+1}]$, Gasparini and Humphreys^15^ showed that the (conditional) density of a random variable W, defined as the time to diagnosis of the first distant metastasis (counting time from detection of the primary tumour), is

(7) $$f_{W|V=v,R=r}(w)=\frac{\sigma }{r}(k_{W}+1){\left(\frac{w}{r}+\log\;\frac{v}{V_{0}}\right)}^{k_{W}}\exp\;\left[-\sigma {\left(\frac{w}{r}+\log\;\frac{v}{V_{0}}\right)}^{k_{W}+1}\right],$$

which is conditional on tumour inverse growth rate r and a volume at detection V=v. We extend their model to allow for between-subject heterogeneity, analogously to the model from Section 3. Specifically, we assume that the spread parameter σ follows a Gamma distribution with shape $\omega_{1}$ and inverse scale $\omega_{2}$. As in Gasparini and Humphreys, we make the assumptions that metastatic seeding completely stops at diagnosis of the primary tumour, and that already seeded, successful colonies are not affected by surgery following diagnosis/treatment, and that the times from seeding to detection are the individual specific times $t_{0}$.^15^ We can then re-formulate the density function for time to detection of distant metastasis W as:

(8) $$f_{W|V=v,R=r}(w)=\frac{k_{W}+1}{r}{\left(\frac{w}{r}+\log\;\frac{v}{V_{0}}\right)}^{k_{W}}\frac{\omega_{1}{\omega_{2}}^{\omega_{1}}}{{\left[\omega_{2}+{\left(\frac{w}{r}+\log\;\frac{v}{V_{0}}\right)}^{k_{W}+1}\right]}^{\omega_{1}+1}},$$

for all values $0\leq w\leq r\log\;(V_{0}/V_{\text{Cell}})$; for $w>r\log\;(V_{0}/V_{\text{Cell}})$, the density is null. The survival function for time to detection of distant metastasis follows as:

(9) $$S_{W|V=v,R=r}(w)=\left\{ \begin{array}{cc} {\left\{\omega_{2}/\left[\omega_{2}+{\left(\frac{w}{r}+\log\;\frac{v}{V_{0}}\right)}^{k_{W}+1}\right]\right\}}^{\omega_{1}} & \text{if}\;0\leq w\leq r\log\;(V_{0}/V_{\text{Cell}})\\ {\left\{\omega_{2}/\left[\omega_{2}+{\left(\log\;\frac{v}{V_{\text{Cell}}}\right)}^{k_{W}+1}\right]\right\}}^{\omega_{1}} & \text{if}\;w>r\log\;(V_{0}/V_{\text{Cell}}) \end{array}\right.$$

Interestingly, under our modelling assumptions, $\{\omega_{2}/[\omega_{2}+(\log\;(v/V_{\text{Cell}}){)}^{k_{W}+1}]{\}}^{\omega_{1}}$ can be considered as a (conditional) cure fraction as it represents *the (conditional) probability that a woman will never be diagnosed with distant metastasis*. We show later how this proportion can be estimated conditional on mode of detection (screening vs. symptomatic), tumour volume, and number of affected lymph nodes at diagnosis (Section 10).

Finally, for a tumour of volume v, we can formulate the probability of having detected metastasis at the time of diagnosis, as:

(10) $$P(W\leq 0|V=v)=1-\frac{{\omega_{2}}^{\omega_{1}}}{{\left[\omega_{2}+{\left(\log\;\frac{v}{V_{0}}\right)}^{k_{W}+1}\right]}^{\omega_{1}}}$$

## 5 Joint modelling of local and distant metastatic spread

In Sections 6 and 7, we derive likelihood functions for the joint probability of tumour size, lymph node spread, and time to distant metastases. Because we assume that detection is a function of tumour size (and not lymph node or distant metastatic spread) it is convenient to work with the joint distribution of the number of affected lymph nodes N=n and the time to first detected distant metastasis W=w, given the size of the tumour at detection (either symptoms or screening), V=v, and the inverse growth rate R=r:

(11) $$f_{N,W|V=v,R=r}(n,w)$$

There are several ways to connect the two processes, that is, spread to the lymph nodes and distant metastatic spread. For instance, the two processes could be connected by specifying correlated rates of spread; there are though computational difficulties associated with this approach. We instead take a copula modelling approach: this is convenient since we have already specified the marginal distributions of N and W (Sections 3 and 4), and is reasonable in the absence of a clear underlying biological model.^16,17^ A copula is defined as a multivariate cumulative distribution function (CDF) for which the marginal probability distributions are uniform on the interval [0,1]. Formally, if F is a bivariate CDF with univariate CDF margins $F_{1},F_{2}$ then, according to Sklar’s theorem,^18^ for every bivariate distribution there exists a copula representation such that

(12) $$F(x_{1},x_{2}|\theta )=C(F_{1}(x_{1}),F_{2}(x_{2});\theta )$$

for a certain parameter (or vector of parameters) θ. In our setting, let C be a bivariate copula and $F_{N|V=v,R=r}(n)$ and $F_{W|V=v,R=r}(w)$ be the CDFs of affected lymph nodes at detection and time to distant metastasis, respectively. The joint bivariate cumulative distribution can therefore be defined using the copula C as

(13) $$F_{N,W|V=v,R=r}(n,w)=C(F_{N|V=v,R=r}(n),F_{W|V=v,R=r}(w))$$

This holds for any copula function C, with possible choices being discussed in Section 8. Note that we omit θ from equation (13) and onwards, in order to simplify our notation. Furthermore, we assume θ⊥⊥v,r; the CDFs in the copula function are however conditional on v and r.

The joint bivariate density function follows as:

(14) $$f_{N,W|V=v,R=r}(n,w)=\frac{\partial^{2}\;C(F_{N|V=v,R=r}(n),F_{W|V=v,R=r}(w))}{\partial n\;\partial w}$$

Note that (14) is a mixed bivariate distribution, with a discrete margin (the number of affected lymph nodes) and a continuous margin (the time to detection of distant metastasis). To simplify calculations, we discretise the continuous margin to obtain a bivariate discrete distribution; we, therefore, consider discrete time from here on.

We note also that the copula C is guaranteed to be unique only if the margins are continuous; with discrete margins, many copula functions are possible.^19,20^ Nevertheless, copula models for discrete distributions are still valid constructions that are uniquely defined on Ran(N)×Ran(W), with Ran(X) representing the range of margin X. Therefore, identification concerns for a copula with discrete margins diminish as the margins completely cover the outcome domains, which is likely to happen in our case, as we discretise time.

## 6 Likelihood function in the absence of screening

In this section, we describe a likelihood function for the joint model introduced in Section 5. We do this first in the absence of screening, as an intermediate step, before developing the algorithm that we will use in our observational data analysis (with screening incorporated). The contribution to the likelihood differs between observed events (patients who are diagnosed with distant metastasis during follow-up), left-censored subjects (with detected metastasis at diagnosis), and right-censored subjects, who are event-free at the end of follow-up.

The likelihood contribution for observed events ( w>0) is defined as

(15) $$L=f_{V_{\text{det}}}(v)\int_{R}P(N=n,W=w|V_{\text{det}}=v,R=r)f_{R|V_{\text{det}}=v}(r)\;dr$$

where

(16) $$\begin{array}{cc} & P(N=n,W=w|V_{\text{det}}=v,R=r)\\ & =\left\{ \begin{array}{cc} C(F_{N|v,r}(0),F_{W|v,r}(w))-C(F_{N|v,r}(0),F_{W|v,r}(w-1)) & \text{if}\;n=0\\ C(F_{N|v,r}(n),F_{W|v,r}(w))-C(F_{N|v,r}(n-1),F_{W|v,r}(w))\\ \;\;-C(F_{N|v,r}(n),F_{W|v,r}(w-1))+C(F_{N|v,r}(n-1),F_{W|v,r}(w-1)) & \text{if}\;n=1,2,\;...\;\ldots \end{array}\right. \end{array}$$

Note that, from here on, $F_{N|v,r}\equiv F_{N|V=v,R=r}$ and $F_{W|v,r}\equiv F_{W|V=v,R=r}$, to simplify our notation.

The likelihood contribution for left-censored observations is defined as

(17) $$L=f_{V_{\text{det}}}(v)P(N=n,W\leq 0|V_{\text{det}}=v)$$

where

(18) $$\begin{array}{cc} P & \left(N=n,W\leq 0|V_{\text{det}}=v\right)\\ & =\left\{ \begin{array}{cc} C(F_{N|v,r}(0),F_{W|v,r}(0)) & \text{if}\;n=0\\ C(F_{N|v,r}(n),F_{W|v,r}(0))-C(F_{N|v,r}(n-1),F_{W|v,r}(0)) & \text{if}\;n=1,2,\;...\;\ldots \end{array}\right. \end{array}$$

where $F_{W|v,r}(0)$ follows from equation (10). Note that this contribution does not require integrating over the distribution of inverse growth rates, given that all components in (18) are independent of R.

Finally, the likelihood contribution for right-censored observations is defined as

(19) $$L=f_{V_{\text{det}}}(v)\int_{R}P(N=n,W>w|V_{\text{det}}=v,R=r)f_{R|V_{\text{det}}=v}(r)\;dr$$

where

(20) $$\begin{array}{cc} P & \left(N=n,W>w|V_{\text{det}}=v,R=r\right)\\ & =\left\{ \begin{array}{cc} C(F_{N|v,r}(0),1)-C(F_{N|v,r}(0),F_{W|v,r}(w)) & \text{if}\;n=0\\ C(F_{N|v,r}(n),1)-C(F_{N|v,r}(n),F_{W|v,r}(w))\\ \;-C(F_{N|v,r}(n-1),1)+C(F_{N|v,r}(n-1),F_{W|v,r}(w)) & \text{if}\;n=1,2,\;...\;\ldots \end{array}\right. \end{array}$$

The three possible contributions to the likelihood function are for a given copula function C, and require integrating out the distribution of inverse growth rates conditional on tumour size at detection, $f_{R|V_{\text{det}}=v}(r)$, which, for the tumour growth and symptomatic detection models used here, has been shown by Isheden and Humphreys^13^ to be equal to

(21) $$f_{R|V_{\text{det}}=v}(r)=\frac{\left[\tau_{2}+\eta (v-V_{0})\right]}{\Gamma (\tau_{1}+1)}\{r[\tau_{2}+\eta (v-V_{0})]{\}}^{(\tau_{1}+1)-1}\exp\;\{-r[\tau_{2}+\eta (v-V_{0})]\}$$

In practice, we approximate the integral using numerical integration. In Section 9 of this article and Section A.1 of the Supplemental Material available online, we describe a Monte Carlo simulation study which we carried out to validate the estimation procedure described above, together with our implementation, based on using a bivariate Frank copula.

## 7 Likelihood function for a screened population

We now formulate the likelihood function for a screened population. We assume that, for all incident cases patients, information is available on whether their primary tumour was detected by screening or via symptoms, and that dates of their (negative) screens prior to diagnosis are also known (as is the case for the participants of the study presented in Section 11). Individual contributions to the likelihood will be based on the joint probability of tumour size, number of affected lymph nodes, and time to distant metastasis, conditional on mode of detection and dates of prior negative screens.

We start by defining a model for screening sensitivity S, which is assumed to follow a logistic function given covariates X and model parameters β:

(22) $$S(X|\beta )=\frac{\exp\;(X\beta )}{1+\exp\;(X\beta )}$$

The linear predictor Xβ includes an intercept, and X (in our case) corresponds to the diameter of the tumour. We further assume that only tumours with a diameter larger than $d_{0}=0.5$ mm are detectable via screening, or equivalently, that screening sensitivity $S(d\leq d_{0})=0$.

The screening sensitivity is then used, together with the sub-models introduced in the previous section, to formulate the likelihood function. In essence, detection via screening and via symptoms act as competing events. We note that in the derivations below, we rely on stable disease assumptions (described by Isheden and Humphreys^13^ ) to formulate the likelihood function, and we use notation that is similar to that of previous work^13,15^ :

-
A is used to denote that there is a (yet to be detected) tumour in a woman’s breast at a specified point in time;
-
$B_{0}$ is used to denote that a tumour is screen-detected;
-
$B^{c}=B_{1}^{c}\cap B_{2}^{c}\cap \cdots \cap B_{p}^{c}$ is used to denote a series of time-ordered p negative screens that occurred prior to detection of a tumour.

Assuming all of the above, the likelihood contribution for screen-detected ( SD) tumours is defined as:

(23) $$L^{SD}\propto P(B_{0}|V=v)P(V=v,N=n,W=w|A)P(B^{c}|A,V=v,N=n,W=w),$$

where V is a random variable representing the tumour volume at the time of screen detection with observed value v. The term for screening history, $P(B^{c}|\cdots )$, can be omitted for women with no history of negative screens.

The term P(V=v,N=n,W=w|A) represents the joint probability of tumour size, distant, and loco-regional metastasis given the presence of a tumour in the breast. This can be re-written as

(24) P(V=v,N=n,W=w|A)=P(N=n,W=w|V=v)P(V=v|A)

and, using Theorem 2 from Isheden and Humphreys,^13^ this becomes

(25) $$P(V=v,N=n,W=w|A)\propto P(N=n,W=w|V=v)P(V_{\text{det}}=v),$$

where $P(V_{\text{det}}=v)$ is defined in equation (3). The screening history term in (23) can be re-written as

(26) $$\begin{array}{cc} P(B^{c} & \left|A,V=v,N=n,W=w\right)\\ & =\int_{R}\left[\prod_{q=1}^{\;p}P(B_{q}^{c}|R=r,V=v)\right]f_{R|V=v,N=n,W=w,A}(r)\;dr \end{array}$$

where the conditional distribution of inverse growth rates within the integral can be re-written as

(27) $$f_{R|V=v,N=n,W=w,A}(r)=\frac{\;f_{N,W|V=v,R=r}(n,w)f_{R|V=v,A}(r)}{f_{N,W|V=v}(n,w)}$$

According to Theorem 3 in Isheden and Humphreys,^13^ we can write

(28) $$f_{R|V=v,A}(r)=f_{R|V_{\text{det}}=v}(r)$$

Therefore, the likelihood can be finally written as:

(29) $$\begin{array}{cc} L^{SD} & \propto \;P(B_{0}|V=v)\times P(V_{\text{det}}=v)\\ & \times \;\int_{R}\left[\prod_{q=1}^{\;p}P(B_{q}^{c}|R=r,V=v)\right]f_{N,W|V=v,R=r}(n,w)f_{R|V_{\text{det}}=v}(r)\;dr \end{array}$$

For women that are detected symptomatically ( SYM), the likelihood can be written as

(30) $$L^{SYM}\propto P(V_{\text{det}}=v,N=n,W=w|A)P(B^{c}|A,V_{\text{det}}=v,N=n,W=w),$$

It can be shown, using analogous arguments, that, for cancers detected symptomatically, the likelihood contribution can be re-written as in (29) but with the first term being omitted:

(31) $$\begin{array}{cc} L^{SYM} & \propto \;P(V_{\text{det}}=v)\;\times \\ & \int_{R}\left[\prod_{q=1}^{\;p}P(B_{q}^{c}|R=r,V=v)\right]f_{N,W|V=v,R=r}(n,w)f_{R|V_{\text{det}}=v}(r)\;dr \end{array}$$

As in the absence of screening, the contribution will once again differ between observed events, left-censored and right-censored observations; possible values for the bivariate density $f_{N,W|R=r,V=v}(n,w)$ are described in Section 6, and we again discretise the bivariate distribution for simplicity. The first term within the integrals in (29) and (31) is the (conditional) probability of p negative screens, which can be calculated using backwards projection, with the procedure described in more detail elsewhere.^12–15^ Numerical integration, to average over the conditional distribution of inverse growth rates $f_{R}(r|V_{\text{det}}=v)$, is once again required. As in previous work,^13,15^ and given that we discretise the continuous margin (i.e. time) to obtain a bivariate discrete distribution (see for reference Section 5), it is relatively straightforward to calculate the above likelihood functions which, as written above, are known only up to a proportionality constant. For instance, considering observed events for screen-detected women as an example, we need to first calculate equation (29) (where $f_{N,W|V=v,R=r}(n,w)$ follows from equation (16)) and then calculate the same quantity but with the complementary of $f_{N,W|V=v,R=r}(n,w)$ instead, for example, one minus equation (16) in this case. Luckily, the computational overhead of this procedure is only marginal.

As in the absence of screening (Section 6), we carried out a simulation study to validate the estimation procedure (and our implementation) in the presence of screening, based on using a Frank copula. This is described in brief in Section 9 and in full in Section A.2 of the Supplemental Material.

## 8 Choice of the copula function

In principle, different copula functions C are possible; however, some formulations have advantageous properties. Specifically, we focus here on Archimedean copulae: they admit explicit formulae for the bivariate case and allow modelling dependence with a single parameter, θ, governing the strength of the association between the two components. The Archimedean copulae that we consider are described in Table 1.

**Table 1.** Most important Archimedean copulae, including their bivariate formulation, the domain of the association parameter θ, and possible correlation values (in terms of Kendall’s τ) that could be represented. Note that the product copula is also known as the *independence* copula.

Name of copula	Bivariate copula C(u,v;θ)	Domain of θ	Possible correlation τ
Ali-Mikhail-Haq	$\frac{uv}{1-\theta (1-u)(1-v)}$	θ∈[−1,1]	τ∈[−0.18,0.33]
Clayton	$[\max\{u^{-\theta }+v^{-\theta }-1;0\}{]}^{-1/\theta }$	θ∈[−1,∞)∖{0}	τ∈[−1,1]∖0
Frank	$-\frac{1}{\theta }\log\;[1+\frac{\left(\exp\;(-\theta u)-1)(\exp\;(-\theta v)-1\right)}{\exp\;(-\theta )-1}]$	θ∈R∖{0}	τ∈[−1,1]∖0
Gumbel	$\exp\;[-((-\log\;(u){)}^{\theta }+(-\log\;(v){)}^{\theta }{)}^{1/\theta }]$	θ∈[1,∞)	τ∈[0,1]
Product	uv	—	τ=0
Joe	$1-[(1-u{)}^{\theta }+(1-v{)}^{\theta }-(1-u{)}^{\theta }(1-v{)}^{\theta }{]}^{1/\theta }$	θ∈[1,∞)	τ∈[0,1]

It would also be possible to use a Gaussian copula, which is constructed from a multivariate normal distribution using the probability integral transform as follows (for the bivariate case):

(32) $$C_{\text{Gaussian}}(u,v;\theta )=\Phi_{2}\left(\Phi^{-1}(u),\Phi^{-1}(v);\theta \right),$$

where θ is the off-diagonal term of the correlation matrix for the bivariate normal distribution and $\Phi_{2}$ and Φ are the CDFs of a bivariate and univariate standard normal distribution, respectively. Interestingly, in the bivariate case of a Gaussian copula, the correlation between the margins is characterised by a single parameter, as with Archimedean copulae; that is not the case for Gaussian copulae with more than two dimensions. However, using this copula would be computationally demanding as $\Phi_{2}$ does not have a closed-form and would need to be computed numerically.

The choice of the copula function affects the shape of the association structure and the possible correlation values that can be captured and described; for instance, some formulations yield a symmetric association structure (e.g. the Frank and Gaussian copulae), while others do not. Unless prior knowledge about the association structure is available, the choice of the copula structure can be data-driven, for example, using information criteria such as the Akaike information criterion (AIC) and the Bayesian information criteria (BIC) or the observed correlation between the margins.

## 9 Validating the estimation procedure via Monte Carlo simulation

We validated the estimation procedure by designing and running two simulation studies, one in the absence, and one in the presence, of screening; specifically, the aim of these simulation studies was to assess whether our estimation procedure could retrieve the correct values of the model parameters.

We simulated independent datasets, each with 1500 cancer patients, with outcomes simulated under the full joint model described above, both in the presence and absence of screening. We carried out our simulations assuming a Frank copula for simplicity, but we expect our results to generalise to other copulae functions. More details on the settings of the simulation studies are described in the Supplemental Material.

We ran 500 repetitions for the simulation in the absence of screening and 800 repetitions for the simulation for a screened population after preliminary analyses suggested these numbers of repetitions to be sufficient for constraining Monte Carlo errors to 0.01 or less.

The results of our simulations showed (1) that our estimation procedure could retrieve the correct model parameters with no to negligible bias, both in the absence and presence of screening, (2) that estimating the model standard errors using the inverse of the Hessian matrix at the optimum was satisfactory, and that (3) the small, aforementioned bias did not affect coverage probability which was (overall) close to the optimal value of 0.95. As expected, Monte Carlo errors were smaller than 0.01 (overall) showing that the numbers of repetitions were satisfactory for our aims. Further details and results of our simulations are available in the Supplemental Material available online.

## 10 Model-based predictions

After fitting the joint model introduced in Section 5, a variety of useful predictions can be obtained.

First, we can define the probability of having detected distant metastases at diagnosis of the primary tumour given size of the tumour and number of affected lymph nodes:

(33) $$P(W\leq 0|N=n,V=v)=\frac{P(N=n,W\leq 0|V=v)}{P(N=n|V=v)}$$

The numerator follows from equation (18), while the denominator is the probability mass function of the negative binomial distribution from equation (6) (volume at diagnosis can be substituted for $V_{\text{det}}$ in both probabilities since the quantities are independent of R).

Second, we can define the probability of having diagnosed or latent/undiagnosed distant metastases given size of the tumour and number of affected lymph nodes at diagnosis of the primary tumour:

(34) $$P(W\leq w^{\infty }|N=n,V=v)=\frac{P(N=n,W<w^{\infty }|V=v)}{P(N=n|V=v)}$$

The numerator can be calculated starting from equation (20), with $F_{W}(w^{\infty })$ corresponding to the lower part of equation (9); the denominator is once again the probability mass function of the negative binomial distribution of affected lymph nodes. One minus the quantity in equation (34) represents a conditional *cure* probability; this is discussed further in Section 12.

Finally, we can define the survival probability at any time $w^{*}>0$, that is, the probability of remaining free from diagnosed distant metastases up until $w^{*}$, given the size of the tumour and number of affected lymph nodes at diagnosis and conditional on being free of detected distant metastasis at diagnosis of the primary tumour:

(35) $$\begin{array}{cc} & \frac{P(W>w^{*}|N=n,V=v)}{P(W>0|N=n,V=v)}=\frac{P(W>w^{*},N=n|V=v)}{P(W>0,N=n|V=v)}\\ & \;\;=\frac{\int_{R}P(W>w^{*},N=n|V=v,R=r,B^{c})f_{R|V=v}(r)\;dr}{\int_{R}P(W>0,N=n|V=v,R=r,B^{c})f_{R|V=v}(r)\;dr} \end{array}$$

Both numerator and denominator follow from equation (20), while accounting for screening history as described when defining the likelihood function for a screened population in Section 7; this further requires integrating over the conditional distribution of inverse growth rates on tumour volume at detection, which we perform numerically.

## 11 An analysis of data from Swedish postmenopausal breast cancer patients

The model introduced in this manuscript was used to analyse data collected from incident cases in a case-control study of postmenopausal breast cancer in Sweden (CAHRES; Cancer And Hormone REplacement Study).^21^ Data on the participants (women born and residing in Sweden, aged 50–74, diagnosed with an incident primary invasive breast cancer between 1 October 1993 and 31 March 1995) was linked to data from the Swedish Cancer Registry and the Stockholm-Gotland Breast Cancer Registry. The data on distant metastases has been used before to study the association of mammographic density and the risk of distant spread^22^ ; the (marginal) natural history model of lymph node spread was also developed using CAHRES (and other) data.^14,23,24^ Mammographic images and screening histories were collected from mammography screening units and radiology departments, in an extension of the original case-control study; the collection of this data has also been described previously.^25,26^ CAHRES is an old study, which for us has several advantages: first, there is an adequate follow-up to study breast cancer spread to distant metastasis. Second, novel treatments for breast cancer were not available at the time, which may favour some of the basic assumptions of our model.

For this application, we only included women with information on the number of affected lymph nodes and time to distant metastasis, alongside data on tumour size, mode of detection, and screening history. This led to a dataset representing 1581 women, of which 1019 (64.4%) were detected through screening and 562 (35.6%) were detected symptomatically. The median tumour diameter at detection was 15 mm, with an inter-quartile interval of 10–22 mm.

Data for this study was collected before the introduction of sentinel lymph node biopsy; 1091 women (69.0%) had no affected lymph nodes at detection, 170 (10.8%) had one affected lymph node, and 91 (5.8%) had two affected lymph nodes; the remaining 229 women (14.4%) had three or more affected lymph nodes. The 99th percentile of the lymph nodes distribution was 17, and the maximum was 42.

Only one woman had detected distant metastasis at the time of diagnosis of the primary tumour; during follow-up, 288 more women (18.2%) were diagnosed with distant metastasis (Figure B1 in the Supplemental Material available online). Median follow-up time, estimated using the inverse Kaplan-Meier method,^27^ was 5.50 years (95% CI: 5.41–5.59 years). For the estimation of the joint model described in Section 5, we discretised time to diagnosis of distant metastasis in years; as was done in simulations reported in the Supplemental Material available online, the performance of the estimation procedure was found to be acceptable in this setting.

On average, patients with a large number of affected lymph nodes at diagnosis have a higher risk of distant metastasis, with shorter times to detection of distant metastasis compared to patients without lymph node spread. In our data, the estimated Kendall’s τ correlation between the number of affected lymph nodes and the observed times to distant metastasis, using the continuous, observed times, is −0.15. With time discretised in years, the estimated Kendall’s τ is −0.17. Note that these estimates do not take censoring into account. We used this information to choose specific copulae formulations to be tested: we focused on the Frank, Clayton, and Ali-Mikhail-Haq (AMH) copulae as, among the Archimedean copulae introduced in Table 1, these are the only formulations that can accommodate a negative Kendall’s correlation of the observed magnitude. We also fitted a model with a product copula (i.e. assuming independent margins) for comparison purposes.

**Figure 1.**
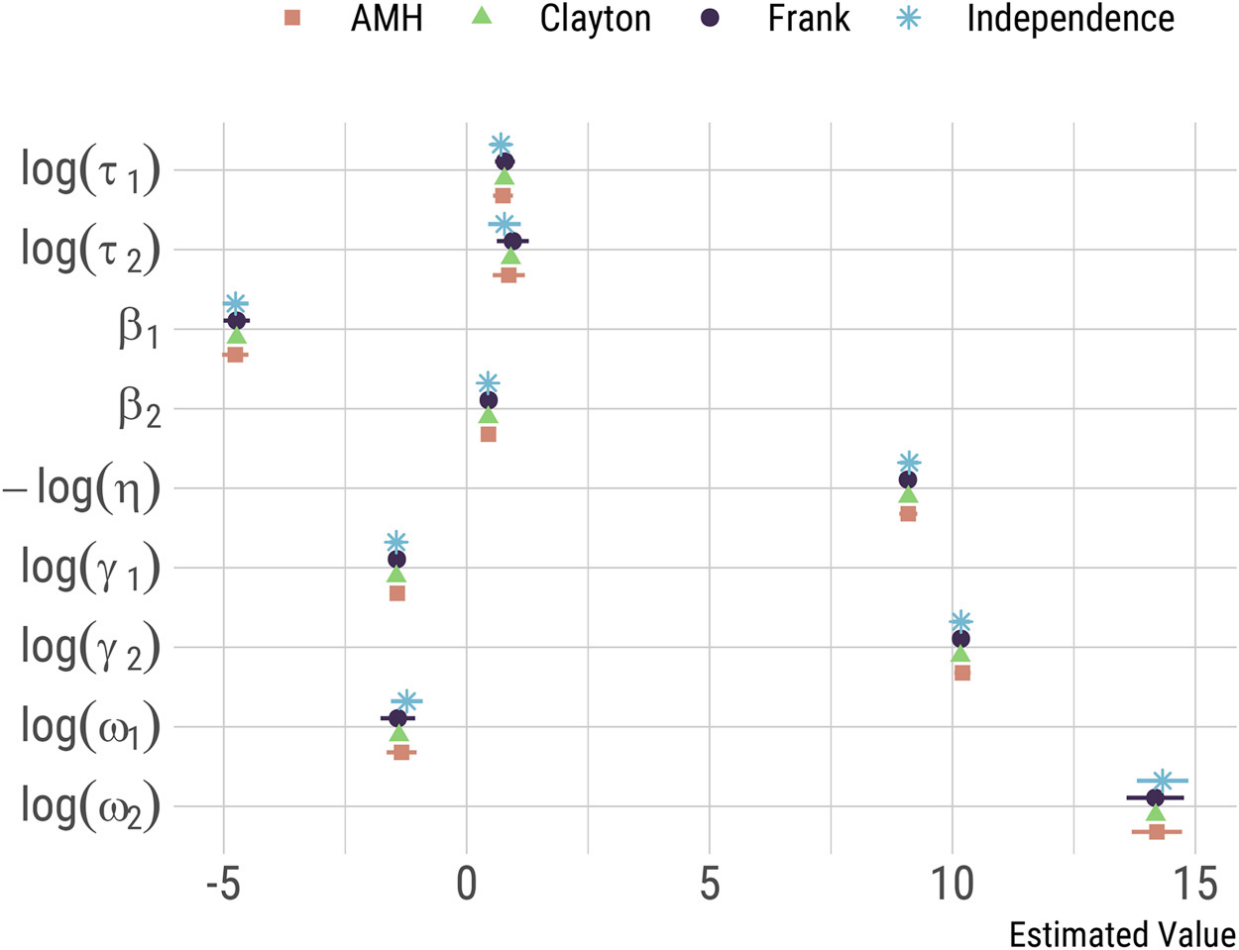
Fitted model coefficients under each copula specification, application to Cancer And Hormone REplacement Study (CAHRES) data. Confidence intervals for the model assuming a Clayton copula are omitted, as the fitted Hessian at the optimum was found to be numerically unstable.

The fitted maximum likelihood values (and model-based estimates of Kendall’s τ coefficients) for the model under each copula formulation are included in Table 2, alongside AIC values. The model with the highest maximum likelihood and the lowest AIC value was the model based on a Frank copula. Point estimates (and confidence intervals) for the parameters of this model are listed in Table 3. Although models based on different copulae performed quite differently, the parameter estimates for the marginal models (of lymph node and distant metastatic spread) were close, as illustrated in Figure 1. Furthermore, the model with the Frank copula provided a significant improvement in fit compared to the model that assumes independent margins (Likelihood ratio test $\chi^{2}=126.24$ with one degree of freedom; $p<10^{-16}$).

**Table 2.** Fitted maximum likelihood values, AIC values, and Kendall’s τ correlations under various copula specifications; application to CAHRES data. 95% confidence intervals for the fitted Kendall’s τ values are calculated using the numerical delta method, given that optimisation was on a different scale, and confidence intervals for the Clayton copula are omitted as inversion of the Hessian was numerically unstable.

	Frank	Clayton	AMH	Independence
Log-likelihood	−6,380.3	−6,417.6	−6,394.9	−6,443.4
AIC	12,780.6	12,855.1	12,809.8	12,904.9
Kendall’s τ	−0.333 ( −0.374 to −0.293)	−0.091	−0.179 ( −0.183 to −0.176)	—

CAHRES: Cancer And Hormone REplacement Study; AMH: Ali-Mikhail-Haq; AIC: Akaike information criterion.

**Table 3.** Fitted parameters (with 95% CI) for the model assuming a Frank copula, application to Cancer And Hormone REplacement Study (CAHRES) data.

Parameter	Fitted value (95% CI)
$\log\;\tau_{1}$	0.788 (0.584 to 0.991)
$\log\;\tau_{2}$	0.950 (0.622 to 1.278)
$\beta_{1}$	−4.734 ( −5.005 to −4.464)
$\beta_{2}$	0.453 (0.406 to 0.500)
−log(η)	9.082 (8.897 to 9.267)
$\log\;\omega_{1}$	−1.416 ( −1.771 to −1.061)
$\log\;\omega_{2}$	14.175 (13.585 to 14.765)
$\log\;\gamma_{1}$	−1.437 ( −1.566 to −1.308)
$\log\;\gamma_{2}$	10.175 (9.997 to 10.352)
θ	−3.304 ( −3.791 to −2.817)

For the best fitting model, we calculated some of the model-based predictions that are described in Section 10. Specifically, we focus here on novel quantities that can be obtained by fitting the model introduced in this manuscript; additional quantities of interest can be calculated, see e.g. previous work.^13–15^ For instance, we estimate from this model a median tumour doubling time of 216 days (95% CI: 209–223), which is comparable to what has been reported elsewhere.^15,28^ Note that standard errors for all model-based predictions reported in this section (such as median doubling time) were estimated using the numerical delta method as implemented in the predictnl function from the rstpm2 package.^29^ Model-based predictions for all copula formulations that were tested are included in the Supplemental Material.

**Figure 2.**
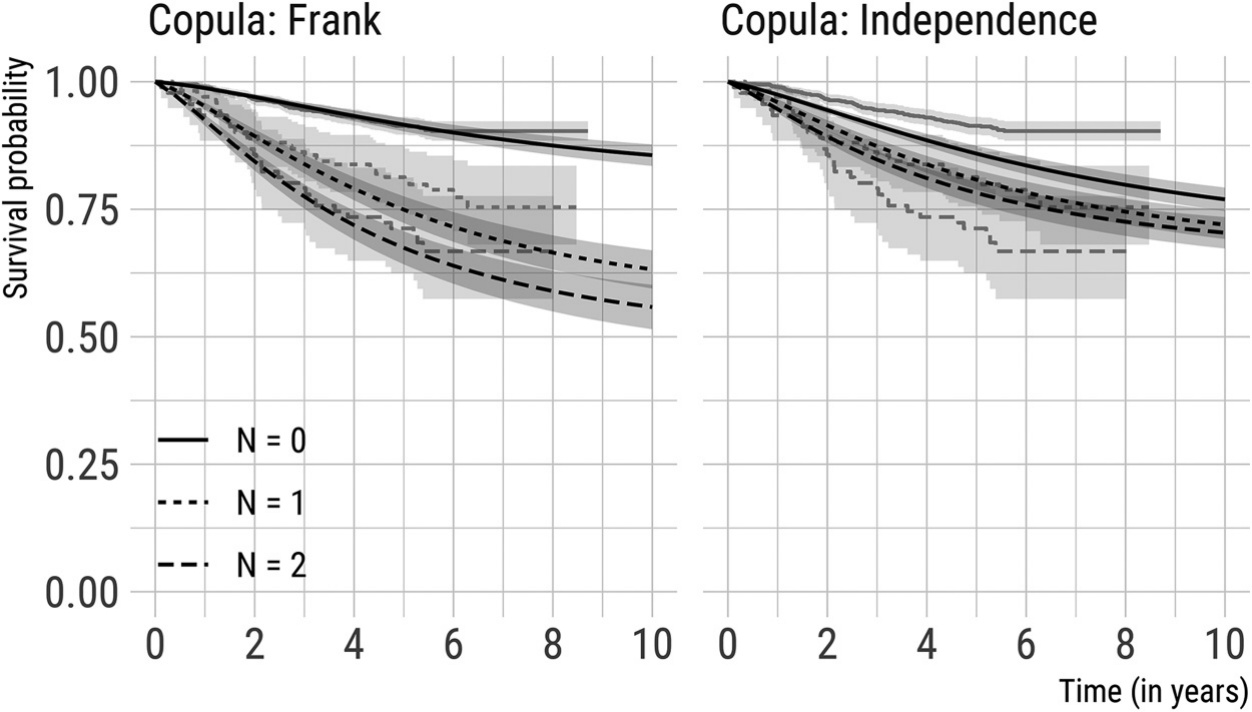
Marginal survival curves (in black) for time to diagnosis of distant metastasis for subjects with zero, one, and two affected lymph nodes at diagnosis. Curves are shown for both the model with a Frank copula and the model with a product (independence) copula and averaged over the observed covariates’ distribution in the Cancer And Hormone REplacement Study (CAHRES). Kaplan-Meier curves (in grey) for relevant subgroups of the study population are included for comparison.

We focus on predictions for the time to distant metastasis, conditional on being free of distant metastasis at the time of diagnosis of the primary tumour. This is relevant since almost all patients have no (detectable) distant metastases at detection of the primary tumour, and is clinically interesting as almost all breast cancer deaths are preceded by a diagnosis of distant metastatic spread.^30^ We first used equation (35) to construct a predicted survival curve for each patient in CAHRES, conditional on their *covariates* (i.e. tumour size, mode of detection, and timing of prior negative screens). We then divided the patients into groups according to the number of affected lymph nodes at diagnosis of the primary tumour. For women with zero, one, and two affected lymph nodes we (separately) marginalised over the observed covariates’ distributions. The resulting curves, for both the model with the Frank copula and the model with the product copula, are depicted (up to 10 years after diagnosis) in Figure 2; 95% point-wise confidence intervals, indicated by shading, are included as well.

Figure 2 also includes Kaplan-Meier curves (depicted in grey), for a reference. Our model-based predictions were reasonably close to the observed data for the model assuming a Frank copula. The other copula formulations showed a poorer fit to the observed data (Figure B2 in the Supplemental Material available online). This illustrates that, although the parameters of the marginal models were estimated to be quite close to each other (Figure 1), the shape of the correlation structure plays an important role in obtaining a good fit to the observed data. As expected, the model assuming independence between the underlying lymph node and distant metastatic spread processes performed less well; the differences in the survival curves across a number of lymph nodes, for the independence copula, are due to a number of affected lymph nodes and time to distant metastases both being dependent on tumour size.

We also estimated the marginal cure fraction, which can be obtained by first calculating one minus equation (34) for every subject in our data, and then taking the average: this yielded an estimated cure probability of 0.697 (95% CI: 0.658–0.736), marginally over the observed distribution of covariates. When averaging, separately, over women with 0, 1 and 2 affected lymph nodes the cure probability estimates were 0.805 (95% CI: 0.772–0.839), 0.553 (95% CI: 0.500–0.605) and 0.479 (95% CI: 0.423–0.535), respectively (Table B2 in the Supplemental Material).

**Figure 3.**
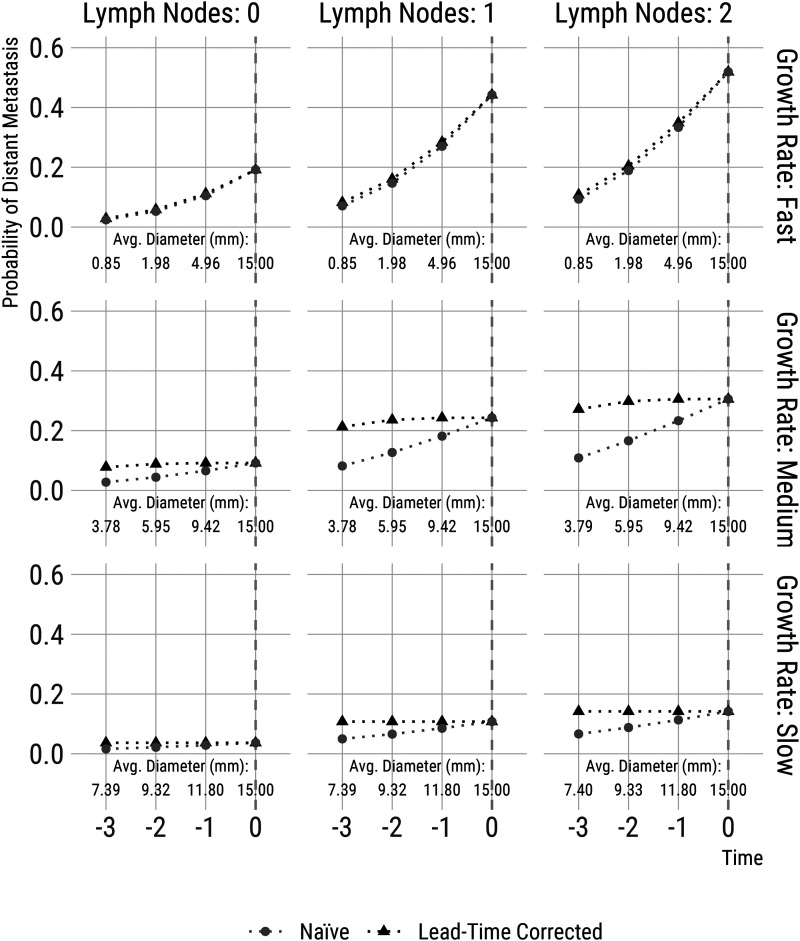
Five-year risks of distant metastasis for women with 15  mm wide tumours, calculated at diagnosis and with risks inferred from scenarios representing early diagnosis, by one, two and three years (Time). Probabilities are estimated using a simulation approach. We simulated 10 million tumours based on the joint model parameter estimates described in Table 3. The *naive* estimates (in grey) are affected by lead-time, while the *lead-time corrected* estimates (in black) are directly comparable to the risk estimates based on detection at 15  mm. Results are presented for tumours with 0, 1 and 2 lymph nodes and by tertiles of inverse growth rates. Each plot includes annotations with average tumour size (as diameter, in mm) at each detection time considered in this microsimulation study.

To illustrate the types of insights that a joint model of lymph node and distant metastatic spread can provide, we performed a microsimulation study to demonstrate the potential consequences of early detection. We simulated 10 million tumours from the inverse growth rate distribution estimated in CAHRES (Table 3) and simulated number of affected lymph nodes at 15 mm, along with a time of detection of distant metastasis (relative to the time the tumour reached 15 mm); this was done using the parameter values from Table 3 for the Frank copula model. For women/tumours without detectable distant metastasis at 15  mm, we then calculated the proportion of women that would have a detected distant metastasis within 5 years of diagnosis of the primary tumour (i.e. from the time the tumour was 15  mm), separately for women with zero, one, or two affected lymph nodes at diagnosis and by tertiles of inverse tumour growth rates (defined according to their fitted distribution). These numbers are represented by the right-most dots in each subplot of Figure 3. Then, we examined what the impact would have been if all tumours had been detected one, two, and three years earlier. Metastases that were seeded after the earlier date of diagnosis would (according to our modelling assumptions) have not been seeded, as the primary tumours would have been removed at this earlier date. As a result, fewer tumours would have distant metastases detected in the future. The five-year risks of distant metastasis counting time from the earlier diagnosis are represented as grey dots in the subplots of Figure 3; these five-year risks are, however, of course, not directly comparable to the five-year risks counting time from when the tumour reached 15  mm. To correct for what is essentially lead-time bias (note that lead time is defined as the time between the early diagnosis, e.g., because of screening, and the time that cancer would have been otherwise diagnosed through symptoms^31–33^ ) we calculated five-year risks from the time the tumour reached 15  mm. These estimates are represented as black triangles in Figure 3; these represent the proportions that should be interpreted (we however keep the naive estimates as a reminder of the importance of incorporating lead time in interpreting early detection in studies of screening). It is clear that early detection substantially improves prognosis only for fast-growing tumours.

**Figure 4.**
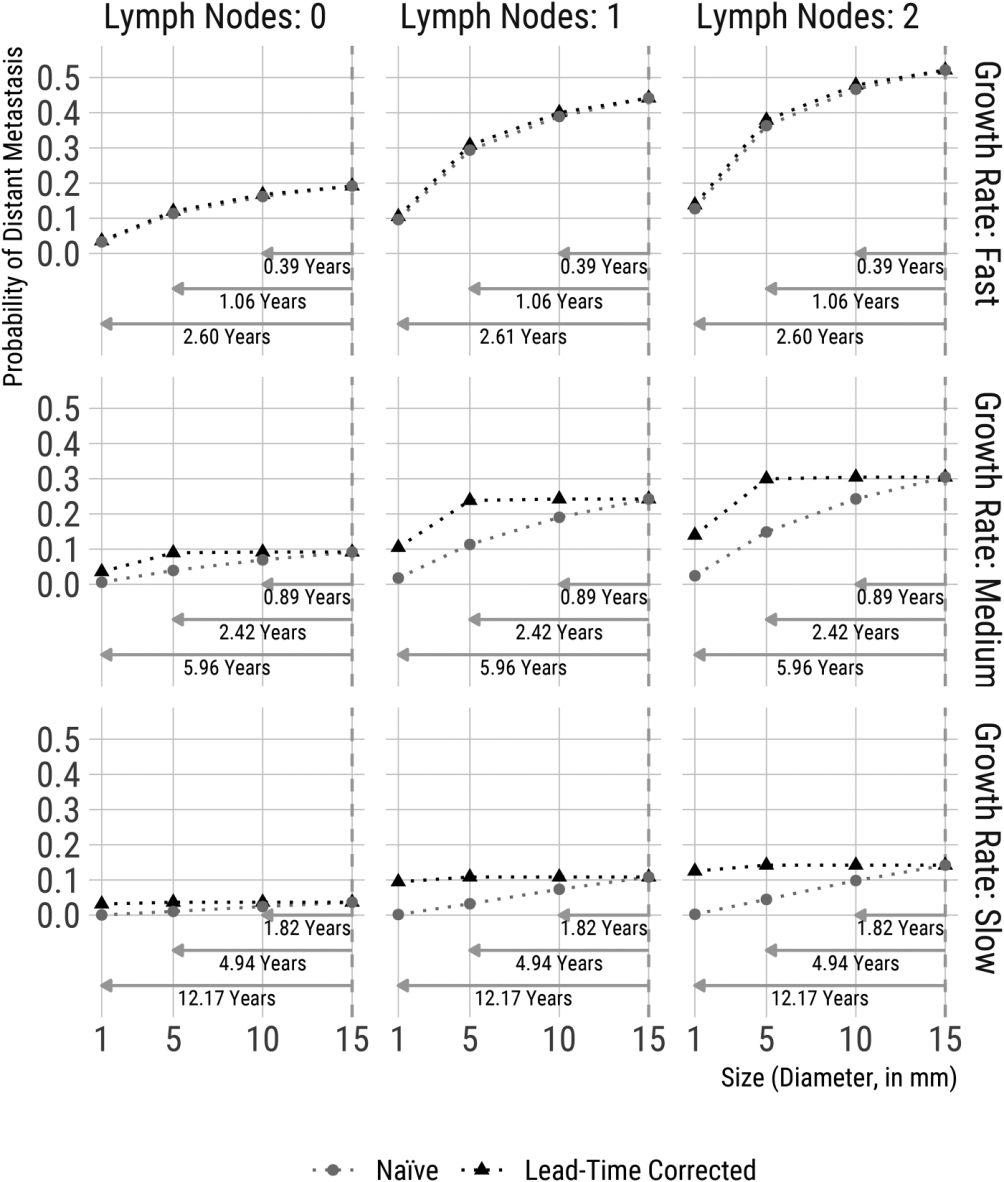
Five-year risks of distant metastasis for women with 15  mm wide tumours, calculated at diagnosis and with risks inferred from scenarios representing early diagnosis at diameters of 10, 5 and 1  mm (Size). Probabilities are estimated using a simulation approach. We simulated 10 million tumours based on the joint model parameter estimates described in Table 3. The *naive* estimates (in grey) are affected by lead-time, while the *lead-time corrected* estimates (in black) are directly comparable to risk estimates based on detection at 15  mm. Results are presented for tumours with 0, 1 and 2 lymph nodes and by tertiles of inverse growth rates. Each plot includes annotations with the average time (in years) it would take to detect tumours at the smaller sizes.

The comparison between growth rate groups with time on the *x*-axis is not entirely straightforward (or fair), since the faster-growing tumours have smaller sizes at any earlier fixed time than the medium and slow-growing tumours. We, therefore, in Figure 4, reformulate the results of our microsimulation example in terms of detection at fixed smaller sizes (thus using tumour diameter for the *x*-axis), to answer, for example, the question: *what would be the risk of distant metastasis if the tumour was detected at a size of 10  mm compared to 15  mm?* Specifically, we go back in time to when the detected tumour would have been 10, 5 and 1  mm wide (compared to 15  mm at diagnosis); at each time, we re-calculate the same probabilities that were described above. We come to the same conclusions when we view the results in these terms; together, the two figures provide a more complete picture of the implications of early detection. Note that average sizes at earlier detection times (in Figure 3) and average times to detect smaller tumours (in Figure 4) are approximately the same across the number of affected lymph nodes after conditioning on growth rates (i.e. row-wise). This is expected from the model for the lymph nodes (Section 3) has the property that the number of lymph nodes is conditionally independent of growth rates, given tumour volume.

## 12 Discussion

Natural history models, such as the one introduced in this manuscript, can provide useful and statistically efficient tools to study the latent history of breast cancer and metastatic spread. An attractive feature is that they explicitly model different aspects of breast cancer (such as growth, detection) using biologically inspired components and assumptions. The joint, copula-based joint model for lymph nodes and distant metastasis that we have developed deviates slightly from this, as copulas are mathematical constructs that do not necessarily have a biological interpretation. We note that evidence showing that direct seeding of tumour cells from the primary tumour to distant organs is the main route of metastatic dissemination has been presented,^34^ but little is known about the dynamic nature of the relationship between lymph node and distant metastasis.

The marginal sub-models that we use through a copula are based on biological concepts,^14,15^ and their interpretations are retained with our approach. Our approach is based on an assumption that the rates of metastatic spread are proportional to the number and rate of cell divisions – in previous work we have been able to demonstrate, in the context of lymph node spread, that this assumption is more consistent with observational data than an assumption that rate of spread is proportional to tumour volume.^14^ The shape of the copula determines a non-linear association between the number of affected lymph nodes and time to distant metastasis, which is consistent with previously reported data.^35^ Overall, the marginal models alone were found to fit observed data reasonably well,^14,15^ therefore we expected the joint model to show a good fit to the data. Results from the applied example in Section 11 aligned with our expectations.

The assumptions for the marginal models have been described in detail before for the lymph nodes model^14^ and for the distant metastasis model.^15^ We also note that assumptions regarding the shape of the association structure (e.g. the parametric form of the copula function) seemed to have a marginal impact on the estimation of the marginal model coefficients (Figure 1). It is important to be clear that our approach is based on somewhat strong assumptions about the effects of tumour removal. An important assumption is that the seeding of distant metastases fully stops when the primary tumour is diagnosed. As discussed by Gasparini and Humphreys,^15^ this will not be fully realistic as treatments administered after a diagnosis of breast cancer (such as surgery followed by chemotherapy and/or radiotherapy) are unlikely to completely stop metastatic seeding. Our modelling assumptions may however be reasonable enough, given the strong evidence supporting the idea that most metastases are seeded early.^36^ Another implicit assumption of our model is that metastases do not cross-seed new metastases; cross-seeding may contribute to faster, or increase heterogeneity in, metastatic growth.^15^ A natural extension of our model consists of, assuming enough data is available, explicitly modelling the efficacy of treatment, for example, by allowing differential efficacy by molecular subtype, or by allowing a fraction of seeded metastasis to survive treatment.

Gasparini and Humphreys^15^ have previously discussed possible extensions to the distant metastasis model; one of these has been dealt with here by generalising and extending the distant metastasis model by including a random effect on the spread parameter σ, which allowed us to derive updated closed-form equations. Other model extensions listed in Gasparini and Humphreys^15^ could be developed in the settings of the joint copula model. To incorporate the inclusion of additional covariates (other than size, mode of detection, and screening history) we could, for example, write the spread parameters in the lymph nodes and distant metastasis submodels as functions of breast cancer subtypes or genetic risk scores. Isheden et al.^24^ illustrate an approach to include covariates in the lymph nodes model as factors that amplify (or reduce) the rate of spread. Lastly, our approach has been developed here specifically for analysing data collected from incident breast cancer cases, for example, in the case-control study that was analysed in Section 11. In future work, we aim to adapt this model to the settings of modern breast cancer screening cohorts.^37^ An alternative approach for studying the recurrence of breast cancer, in terms of time to distant metastasis, is to use multi-state models. Mariotto et al.^6^ recently described such an approach, which they specify as a mixture cure model. Using this approach, and using data from the Surveillance Epidemiology and End Results programme collected between 1992 and 2013, they estimated the probability of metastatic recurrence by age, cancer stage, HR status, and time period of diagnosis. In the current article, we have used our model to study the prognosis of breast cancer patients with given characteristics (size of the primary tumour at detection, mode of detection, screening history, number of affected lymph nodes) in terms of the risk of distant metastasis over time. For instance, in Figure 2, we include survival probabilities for time to diagnosis of distant metastasis, marginalising over the observed distribution of all covariates other than the number of affected lymph nodes. This allowed a direct comparison of the prognosis of breast cancer by affected lymph nodes, based on our joint model, which we showed to be close to the observed data. A benefit of our approach, compared to that of Mariotto et al.,^6^ is that our models for growth and spread are inspired by the biology of breast cancer, thus allowing us to include covariates and make inference on such processes. Both approaches can be used to estimate the cured proportion for women diagnosed with breast cancer. Mariotto et al.^6^ reported cure proportions by HR status, cancer stage at diagnosis, and calendar period of diagnosis; values ranged between 0.29 and 0.88. When averaging over women in our sample, separately for 0, 1 and 2 affected lymph nodes at diagnosis, and assuming the best-fitting copula formulation, we estimated cure proportions to be 0.805, 0.553 and 0.479, respectively, (at 10 nodes this number was down to 0.335). Overall, this proportion was 0.697. This estimate is similar to that reported by Dal Maso et al.^38^ from the EUROCARE-5 study: 0.66 for breast cancers diagnosed in 2000.

The approach introduced in this manuscript can be defined as cause-specific, as we do not explicitly model competing events; thus, we censor study subjects if and when they experience competing events such as death. Despite this, being based on biologically inspired model assumptions, a benefit of our approach is that it still allows inference on the underlying processes of tumour cell spread irrespectively of the occurrence of competing events. For model-based predictions (e.g. of distant metastases), cumulative incidence functions that take into account the competing event of death could be calculated by formulating a second cause-specific model for death and by using the approach described by, for example, Hinchliffe and Lambert.^39^ This would embed our approach within a multi-state modelling framework, that is, allowing us to incorporate competing events such as death (from causes other than breast cancer). In practice, this would mean adding additional states and modelling the relevant transition probabilities. Taking solely a multi-state approach to modelling distant metastases and lymph node spread (where lymph node spread would have to be coarsely discretised) would of course be more straightforward for handling competing risks, but without our natural history modelling component the (standard) multi-state models fail to capture important components such as heterogeneity in growth rates. In future work, we aim to add to our framework a component to explicitly model death due to breast cancer, where distant metastatic spread acts as an intermediate event, and we are currently considering approaches to incorporate death due to other causes.

An additional advantage of our approach is that it can be used to assess how interventions at a population level would impact patient outcomes: we focused on early detection and the risk of distant metastasis, but other interventions can, in principle, be studied, such as personalised mammography screening intervals. Interestingly, our model provided us with a way of estimating the risk of future events while at the same time correcting for lead time. Using our approach we could clearly demonstrate how fast-growing tumours stand to benefit most from early detection.

In conclusion, we have introduced a joint, copula-based model for the latent growth of breast cancer, detection, spread to the lymph nodes, and distant metastatic spread. We have shown that this model was able to capture relevant patterns in data and have demonstrated how, at least under our assumptions, fast-growing tumours stand to gain the most from early detection. Our framework, which can be further extended to better adapt to the complexities of breast cancer progression and treatment, can be fundamentally important for studying the natural history of growth, spread, and prognosis.

## Supplemental Material

sj-pdf-1-smm-10.1177_09622802221122410 - Supplemental material for A natural history and copula-based joint model for regional and distant breast cancer metastasis Click here for additional data file. Supplemental material, sj-pdf-1-smm-10.1177_09622802221122410 for A natural history and copula-based joint model for regional and distant breast cancer metastasis by Alessandro Gasparini and Keith Humphreys in Statistical Methods in Medical Research

## References

[1] Bower H Andersson TML Syriopoulou E, et al. Potential gain in life years for swedish women with breast cancer if stage and survival differences between education groups could be eliminated – three what-if scenarios. Breast 2019; 45: 75–81. 30904700 10.1016/j.breast.2019.03.005

[2] Lee ES Jung SY Kim JY, et al. Identifying the potential long-term survivors among breast cancer patients with distant metastasis. Ann Oncol 2016; 27: 828–833. 26823524 10.1093/annonc/mdw036

[3] Kennecke H Yerushalmi R Woods R, et al. Metastatic behavior of breast cancer subtypes. J Clin Oncol 2010; 28: 3271–3277. 20498394 10.1200/JCO.2009.25.9820

[4] Kennecke H McArthur H Olivotto IA, et al. Risk of early recurrence among postmenopausal women with estrogen receptor-positive early breast cancer treated with adjuvant tamoxifen. Cancer 2008; 112: 1437–1444. 18286526 10.1002/cncr.23320

[5] Putter H van der Hage J de Bock GH, et al. Estimation and prediction in a multi-state model for breast cancer. Biom J 2006; 48: 366–380. 16845902 10.1002/bimj.200510218

[6] Mariotto AB Zou Z Zhang F, et al. Can we use survival data from cancer registries to learn about disease recurrence? the case of breast cancer. Cancer Epidemiol Biomark Prev 2018; 27: 1332–1341. 10.1158/1055-9965.EPI-17-1129 PMC8343992 30337342

[7] Bartoszyński R Edler L Hanin L, et al. Modeling cancer detection: tumor size as a source of information on unobservable stages of carcinogenesis. Math Biosci 2001; 171: 113–142. 11395047 10.1016/s0025-5564(01)00058-x

[8] Tan SYGL van Oortmarssen GJ de Koning HJ, et al. The MISCAN-Fadia continuous tumor growth model for breast cancer. JNCI Monographs 2006; 2006: 56–65. 10.1093/jncimonographs/lgj009 17032895

[9] Plevritis SK Salzman P Sigal BM, et al. A natural history model of stage progression applied to breast cancer. Stat Med 2007; 26: 581–595. 16598706 10.1002/sim.2550

[10] Weedon-Fekjær H Lindqvist BH Vatten LJ, et al. Breast cancer tumor growth estimated through mammography screening data. Breast Cancer Res 2008; 10. 10.1186/bcr2092 PMC2481488 18466608

[11] Weedon-Fekjær H Tretli S Aalen OO. Estimating screening test sensitivity and tumour progression using tumour size and time since previous screening. Stat Methods Med Res 2010; 19: 507–527. 20356856 10.1177/0962280209359860

[12] Abrahamsson L Humphreys K. A statistical model of breast cancer tumour growth with estimation of screening sensitivity as a function of mammographic density. Stat Methods Med Res 2016; 25: 1620–1637. 23839121 10.1177/0962280213492843

[13] Isheden G Humphreys K. Modelling breast cancer tumour growth for a stable disease population. Stat Methods Med Res 2019; 28: 681–702. 29103360 10.1177/0962280217734583

[14] Isheden G Abrahamsson L Andersson T, et al. Joint models of tumour size and lymph node spread for incident breast cancer cases in the presence of screening. Stat Methods Med Res 2019; 28: 3822–3842. 30606087 10.1177/0962280218819568 PMC6745622

[15] Gasparini A Humphreys K. Estimating latent, dynamic processes of breast cancer tumour growth and distant metastatic spread from mammography screening data. Stat Methods Med Res 2022. DOI: 10.1177/09622802211072496. PMC9099158 35103530

[16] Klein CA. Tumour cell dissemination and growth of metastasis. Nat Rev Cancer 2010; 10: 156–156.20094050

[17] Ullah I Karthik GM Alkodsi A, et al. Evolutionary history of metastatic breast cancer reveals minimal seeding from axillary lymph nodes. J Clin Investig 2018; 128: 1355–1370. 29480816 10.1172/JCI96149 PMC5873882

[18] Sklar A. Fonctions de répartition à N dimensions et leurs marges. Publications de l’Institut de Statistique de L’Université de Paris 1959; 8: 229–231.

[19] Genest C Nešlehová J. A primer on copulas for count data. ASTIN Bulletin 2007; 37: 475–515.

[20] Trivedi P Zimmer D. A note on identification of bivariate copulas for discrete count data. Econometrics 2017; 5: 10.

[21] Magnusson C Baron J Persson I, et al. Body size in different periods of life and breast cancer risk in post-menopausal women. Int J Cancer 1998; 76: 29–34. 9533758 10.1002/(sici)1097-0215(19980330)76:1<29::aid-ijc6>3.0.co;2-#

[22] Eriksson L Czene K Rosenberg L, et al. Possible influence of mammographic density on local and locoregional recurrence of breast cancer. Breast Cancer Res 2013; 15. DOI: 10.1186/bcr3450. PMC3979151 23844592

[23] Isheden G Grassmann F Czene K, et al. Lymph node metastases in breast cancer: investigating associations with tumor characteristics, molecular subtypes and polygenic risk score using a continuous growth model. Int J Cancer 2021; 149: 1348–1357. 34097750 10.1002/ijc.33704

[24] Isheden G Czene K Humphreys K. Random effects models of lymph node metastases in breast cancer: quantifying the roles of covariates and screening using a continuous growth model. Biometrics 2021. DOI: 10.1111/biom.13430. 33501643

[25] Rosenberg LU Magnusson C Lindström E, et al. Menopausal hormone therapy and other breast cancer risk factors in relation to the risk of different histological subtypes of breast cancer: a case-control study. Breast Cancer Res 2006; 8. DOI: 10.1186/bcr1378. PMC1413980 16507159

[26] Rosenberg LU Granath F Dickman PW, et al. Menopausal hormone therapy in relation to breast cancer characteristics and prognosis: a cohort study. Breast Cancer Res 2008; 10. DOI: 10.1186/bcr2145. PMC2614511 18803850

[27] Schemper M Smith TL. A note on quantifying follow-up in studies of failure time. Control Clin Trials 1996; 17: 343–346. 8889347 10.1016/0197-2456(96)00075-x

[28] Dahan M Hequet D Bonneau C, et al. Has tumor doubling time in breast cancer changed over the past 80 years? a systematic review. Cancer Med 2021; 10: 5203–5217. 34264009 10.1002/cam4.3939 PMC8335823

[29] Liu XR Pawitan Y Clements M. Parametric and penalized generalized survival models. Stat Methods Med Res 2018; 27: 1531–1546. 27587596 10.1177/0962280216664760

[30] Dillekås H Rogers MS Straume O. Are 90% of deaths from cancer caused by metastases? Cancer Med 2019; 8: 5574–5576. 31397113 10.1002/cam4.2474 PMC6745820

[31] Kramer BS Croswell JM. Cancer screening: the clash of science and intuition. Annu Rev Med 2009; 60: 125–137. 18803476 10.1146/annurev.med.60.101107.134802

[32] Andersson TML Rutherford MJ Humphreys K. Assessment of lead-time bias in estimates of relative survival for breast cancer. Cancer Epidemiol 2017; 46: 50–56. 28027488 10.1016/j.canep.2016.12.004

[33] Abrahamsson L Isheden G Czene K, et al. Continuous tumour growth models, lead time estimation and length bias in breast cancer screening studies. Stat Methods Med Res 2019; 29: 374–395. 30854935 10.1177/0962280219832901

[34] Venet D Fimereli D Rothé F, et al. Phylogenetic reconstruction of breast cancer reveals two routes of metastatic dissemination associated with distinct clinical outcome. EBioMedicine 2020; 56: 102793. 32512508 10.1016/j.ebiom.2020.102793 PMC7281848

[35] Sopik V Narod SA. The relationship between tumour size, nodal status and distant metastases: on the origins of breast cancer. Breast Cancer Res Treat 2018; 170: 647–656. 29693227 10.1007/s10549-018-4796-9 PMC6022519

[36] Hosseini H Obradović MMS Hoffmann M, et al. Early dissemination seeds metastasis in breast cancer. Nature 2016; 540: 552–558. 27974799 10.1038/nature20785 PMC5390864

[37] Strandberg JR Humphreys K. Statistical models of tumour onset and growth for modern breast cancer screening cohorts. Math Biosci 2019; 318: 108270. 31627176 10.1016/j.mbs.2019.108270

[38] Dal Maso L Panato C Tavilla A, et al. Cancer cure for 32 cancer types: results from the EUROCARE-5 study. Int J Epidemiol 2020; 49: 1517–1525. 32984907 10.1093/ije/dyaa128

[39] Hinchliffe SR Lambert PC. Flexible parametric modelling of cause-specific hazards to estimate cumulative incidence functions. BMC Med Res Methodol 2013; 13. DOI: 10.1186/1471-2288-13-13. PMC3614517 23384310

