# Supplementary material for: A natural history and copula-based joint model for regional and distant breast cancer metastasis
Source: Stat Methods Med Res. 2022 Sep 18;31(12):2415–30. doi: 10.1177/09622802221122410 (PMC9703386; doi:10.1177/09622802221122410)
Supplement: sj-pdf-1-smm-10.1177_09622802221122410 - Supplemental material for A natural history and copula-based joint model for regional and distant breast cancer metastasis [file sj-pdf-1-smm-10.1177_09622802221122410.pdf]

Supplementary Material: A Natural History and Copula Based Joint Model for Regional and Distant Breast Cancer Metastasis

June 16, 2022

A Validating the Estimation Procedures

This appendix describes the results of two simulation studies that were run to validate our estimation procedure, for both the model in the absence of screening (section A.1) and the model in the presence of screening (section A.2).

Both simulation studies are coded and run using R version 4.1.1 and analyzed using the `rsimsum` package (R Core Team, 2021; Gasparini, 2018). The likelihood function was optimized using the `optim` function in R and the limited-memory modification of the Broyden-Fletcher-Goldfarb-Shanno (BFGS) quasi-Newton method (Byrd et al., 1995). Standard errors were estimated starting from the estimated Hessian matrix at the optimum, which was numerically approximated using the default procedure provided with the `optim` function in R.

A.1 Validating the Estimation Procedure in the Absence of Screening

A.1.1 Aim

This simulation study aims to assess the ability to retrieve the true data-generating parameters of the model for data collected in the absence of screening.

A.1.2 Data-Generating Mechanisms

We simulated independent datasets, each with 1500 individuals. We assumed that tumours originate from a single spherical cell of volume V_{Cell} corresponding to a diameter of $d_{\text{Cell}} = 0.01$ mm; as described in the manuscript, we assumed that tumours are detectable only once they have reached a given volume V_0 corresponding to a diameter $d_0 = 0.5$ mm. We assumed the parameters $k^N = k^W = 4$ to be fixed.

We simulated inverse growth rates from a Gamma distribution with shape and rate parameters $\tau_1 = \tau_2 = \exp(0.4)$; this corresponds to a Gamma distribution with unit mean and variance of approximately 0.67. We based the choice of these specific parameter values (and those below) on our previous experience of fitting tumour growth models to observational data (Abrahamsson and

Humphreys, 2016; Isheden and Humphreys, 2019; Isheden et al., 2019; Gasparini and Humphreys, 2022).

The tumour volumes at symptomatic detection are simulated using Equation 2 from the manuscript, assuming the parameter $\eta' = -\log(\eta) = 9$.

The bivariate outcome of affected lymph nodes at diagnosis and time to distant metastasis was simulated by assuming a bivariate Frank copula with $\theta = -3$; intuitively, this corresponds to a negative correlation between the two outcomes. We drew random, correlated bivariate samples from the copula function and consequently applied the inversion method to each marginal model to simulate the observed outcomes; here we assumed the parameter values $\log(\gamma_1) = -0.5$, $\log(\gamma_2) = 10$, $\log(\omega_1) = 0$, and $\log(\omega_2) = 15$. Further details on the simulation algorithm for the time to detection of distant metastasis are included elsewhere (Gasparini and Humphreys, 2022).

Finally, we discretized time in years and we applied administrative censoring after 20 years of follow-up. Discretization of time in years can be seen as a worst-case scenario; in practice, discretizing time in finer intervals (e.g. months) should improve the performance of the algorithm.

A.1.3 Estimands

The estimands of interest are the parameters of the model for tumour growth, spread to the lymph nodes, and distant metastatic spread in the absence of screening: $\Psi = \{\tau_1, \omega_1, \omega_2, \gamma_1, \gamma_2, \eta, \theta\}$. In practice, we model τ_1, ω_1, ω_2, γ_1, and γ_2 on the log-scale and $\eta' = -\log(\eta)$.

A.1.4 Methods

We fitted the true data-generating model for data collected in the absence of screening, as described in the main body of the manuscript, to each independently simulated data set. Standard errors for the model parameters were computed by inverting the Hessian matrix at the optimum.

A.1.5 Performance Measures and Number of Replications

The key performance measure of interest is bias, to quantify whether the estimation procedure can retrieve the true data-generating parameters, on average; in order to quantify the uncertainty in the bias estimation, we report Monte Carlo standard errors as well. We also report empirical and model-based standard errors, including relative percentage errors in standard errors, and coverage probability. Performance measures are described in more detail elsewhere (Morris et al., 2019).

To estimate the key performance measure with a given precision, we first ran 10 replications of the simulation study. Allowing a Monte Carlo standard error for bias of 0.01, we would require $n_{\text{sim}} = \text{Var}/\text{MCSE}^2 \approx 415$ repetitions of our full simulation study. This calculation was based on assuming Var $= 0.041542$, which was taken as the largest value among all empirical and model-based standard errors for $\widehat{\Psi}$ obtained from the above-mentioned 10 replications. To be conservative we rounded up the number of repetitions to the nearest 100, which yielded a total of $n_{\text{sim}} = 500$.

Figure A1: Bias with 95% confidence intervals based on the Monte Carlo standard errors, simulation study for the model in the absence of screening.

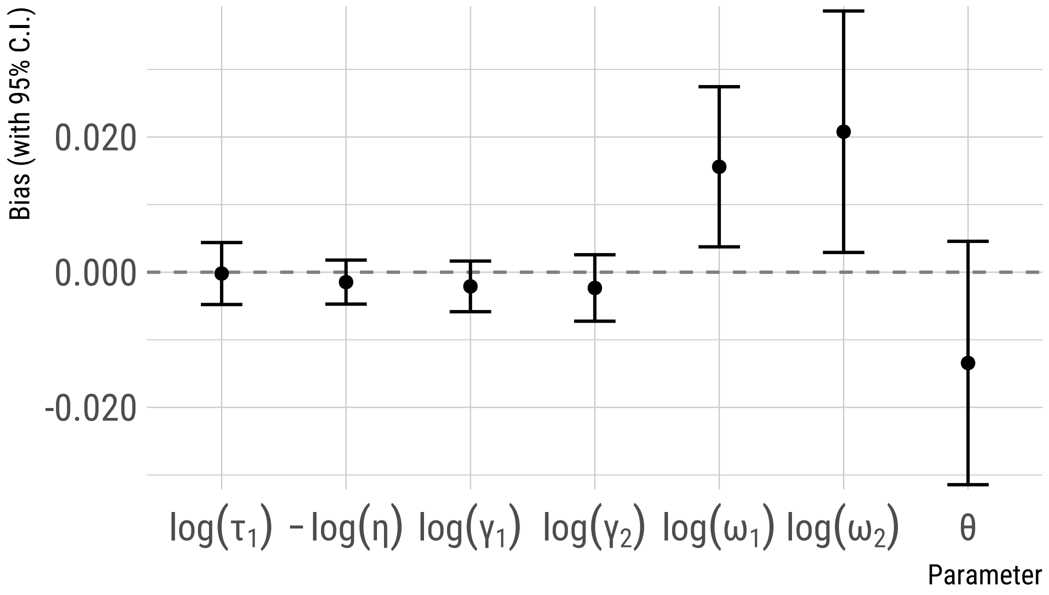

A.1.6 Results

Bias with 95% confidence intervals (based on Monte Carlo standard errors) for each model parameter is depicted in Figure A1. All parameters could be estimated with virtually no bias, except for $\log(\omega_1)$ and $\log(\omega_2)$ (parameters of the marginal model for time to detection of distant metastasis) where we could see a small positive bias in absolute terms. Nevertheless, we deemed this bias to be negligible as the relative bias on the original scale of the parameters was close to zero (Figure A2). It can also be shown that estimation of the model standard errors using the inverse of the Hessian matrix at the optimum yielded satisfactory results, as highlighted by empirical and model-based standard errors being close to each other (Figure A3) and by the small relative percentage error in standard errors (Figure A4).

Finally, we could show that the small, aforementioned bias did not affect coverage probability, which was (overall) close to the optimal value of 95% (Figures A5).

Estimated values for bias, empirical and model-based standard errors, relative percentage errors, and coverage probability, together with their Monte Carlo standard errors, are tabulated for reference in Table A1.

Figure A2: Relative bias with inter-quartile intervals, for parameters on the original scale. Simulation study for the model in the absence of screening.

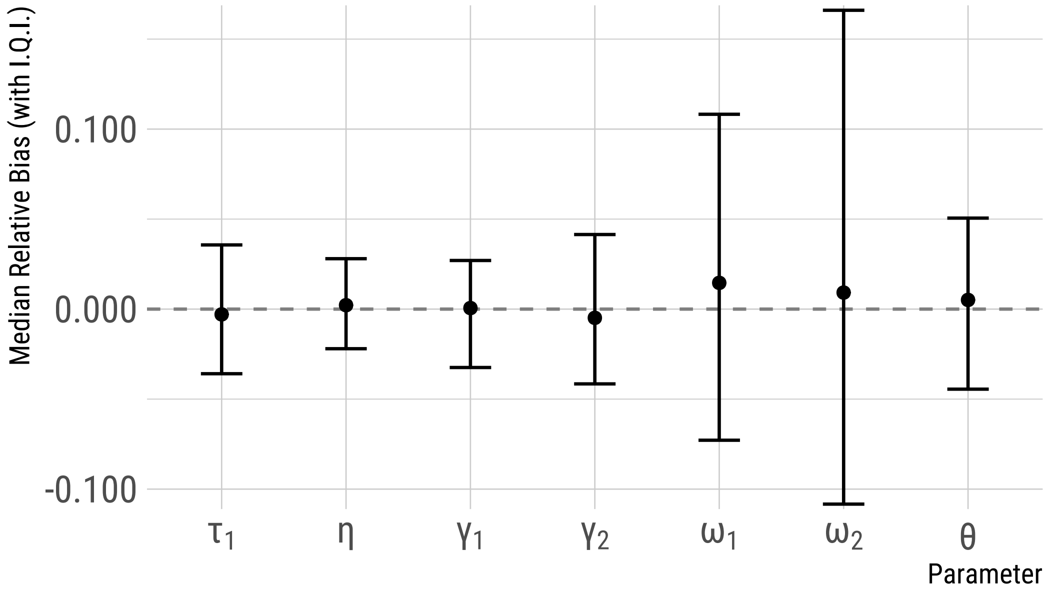

Figure A3: Empirical standard errors and model-based standard errors with 95% confidence intervals based on the Monte Carlo standard errors, simulation study for the model in the absence of screening.

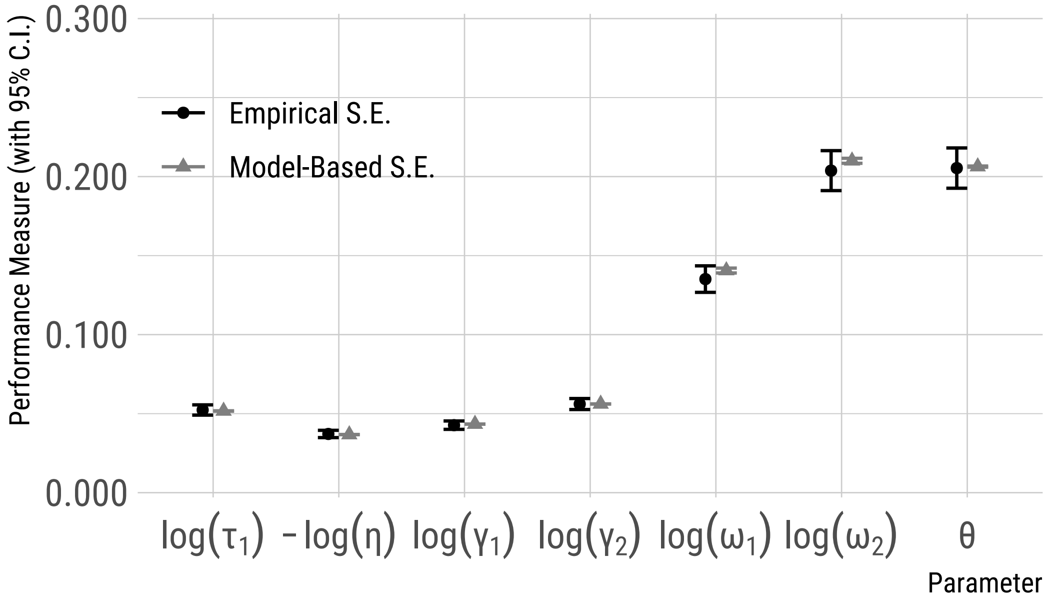

Figure A4: Relative percentage error in standard error with 95% confidence intervals based on the Monte Carlo standard errors, simulation study for the model in the absence of screening.

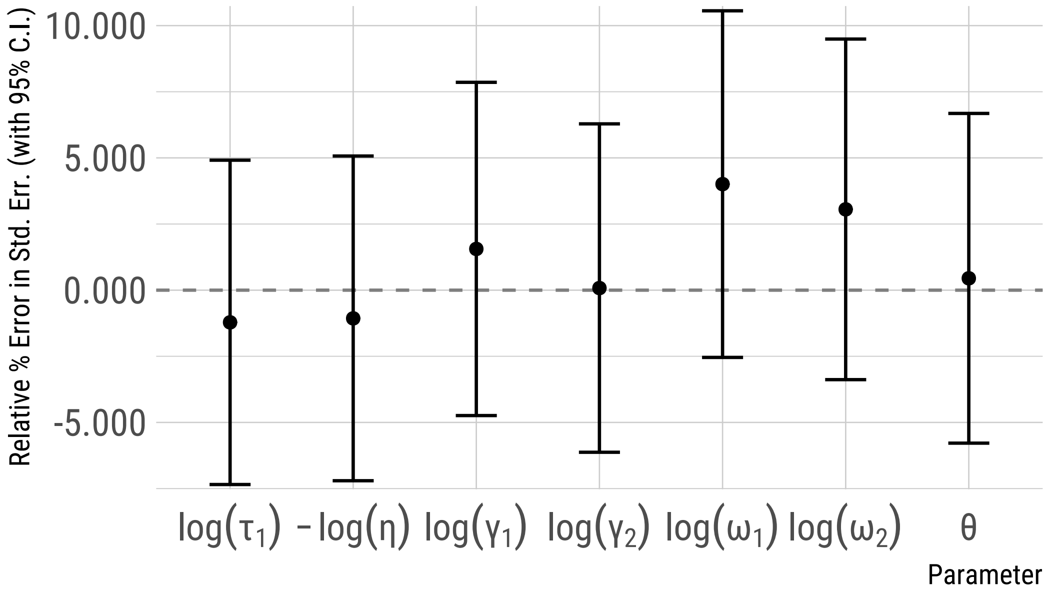

Figure A5: Coverage probability with 95% confidence intervals based on the Monte Carlo standard errors, simulation study for the model in the absence of screening.

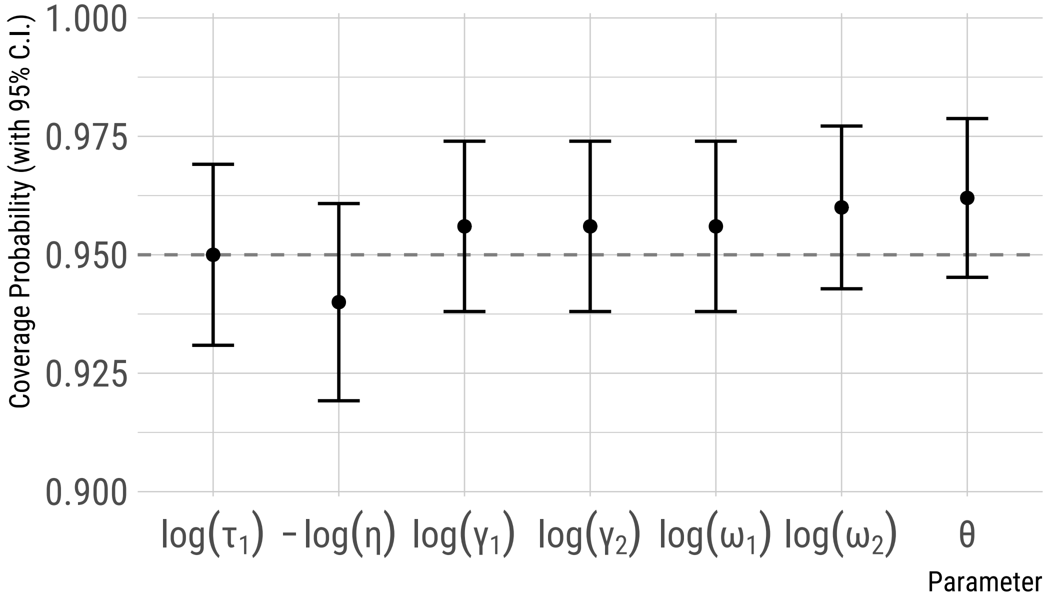

Table A1: Summary statistics for the simulation study to evaluate the estimation procedure for the model in the absence of screening. Values are point estimates with Monte Carlo standard errors in parentheses.

Parameter	Bias	Emp. S.E.	Model S.E.	Relative Error	Coverage
$-\log(\eta)$	-0.001 (0.002)	0.037 (0.001)	0.037 (0.000)	-1.066 (3.131)	0.940 (0.011)
$\log(\gamma_1)$	-0.002 (0.002)	0.043 (0.001)	0.043 (0.000)	1.558 (3.213)	0.956 (0.009)
$\log(\gamma_2)$	-0.002 (0.003)	0.056 (0.002)	0.056 (0.000)	0.079 (3.167)	0.956 (0.009)
$\log(\omega_1)$	0.016 (0.006)	0.135 (0.004)	0.141 (0.001)	4.008 (3.343)	0.956 (0.009)
$\log(\omega_2)$	0.021 (0.009)	0.204 (0.006)	0.210 (0.001)	3.055 (3.284)	0.960 (0.009)
$\log(\tau_1)$	-0.000 (0.002)	0.052 (0.002)	0.052 (0.000)	-1.217 (3.128)	0.950 (0.010)
θ	-0.013 (0.009)	0.205 (0.007)	0.206 (0.000)	0.449 (3.179)	0.962 (0.009)

A.2 Validating the Estimation Procedure for a Screened Population

A.2.1 Aim

This simulation study aims to assess the ability to retrieve the true data-generating parameters of the model for data collected in a screened population.

A.2.2 Data-Generating Mechanisms

The data-generating mechanism for this simulation study is closely related to that described in Section A.1. We, once again, simulated independent datasets of 1500 individuals each, assuming spherical tumours that originate from a single cell of volume V_{Cell} with $d_{\text{Cell}} = 0.01$ mm and that such tumours are detectable only once they cross the threshold of volume V_0 (corresponding to a diameter $d_0 = 0.5$ mm). We again assumed the parameters $k^N = k^W = 4$ to be fixed.
Compared to the previous simulation study, we do not require the constraint $\tau_1 = \tau_2$ (for identifiability purposes) anymore. We, therefore, simulate inverse growth rates from a Gamma distribution with shape parameter $\tau_1 = 2$ and rate $\tau_2 = 4$; this leads to a distribution with a mean of 0.5 and a variance of 0.125.
For tumour volumes at symptomatic detection, we assume again that $\eta' = -\log(\eta) = 9$.
Age at onset of breast cancer was simulated assuming age-increasing rates based on a procedure described previously (Abrahamsson and Humphreys, 2016; Isheden and Humphreys, 2019; Isheden et al., 2019), which use published breast cancer incidence rates (Forastero et al., 2010). We then enforced a screening program starting at the age of 40, with screens every two years. Death was not included in our simulations, analogously as with previous work, see e.g. Isheden et al. (2019); Gasparini and Humphreys (2022). Tumours were detected according to the logistic screening sensitivity function described in the manuscript, including the diameter of the tumour as a covariate and assuming parameters $\beta_1 = -5$ (model intercept) and $\beta_2 = 0.5$ (coefficient of size).
The remaining details of the data-generating mechanisms (including assumed parameters) are analogous to what was previously described in Section A.1.

A.2.3 Estimands

The estimands of interest are the parameters of the model for a screened population: $\Psi = \{\tau_1, \tau_2, \omega_1, \omega_2, \gamma_1, \gamma_2, \beta_1, \beta_2, \eta, \theta\}$. In practice, we model τ_1, τ_2, ω_1, ω_2, γ_1, and γ_2 on the log-scale and $\eta' = -\log(\eta)$.

A.2.4 Methods

The method of interest is the true data-generating model for a screened population, which we fit to each independently simulated data set; once again, standard errors for the model parameters were computed by inverting the Hessian matrix at the optimum.

Figure A6: Bias with 95% confidence intervals based on the Monte Carlo standard errors, simulation study for the model in a screened population.

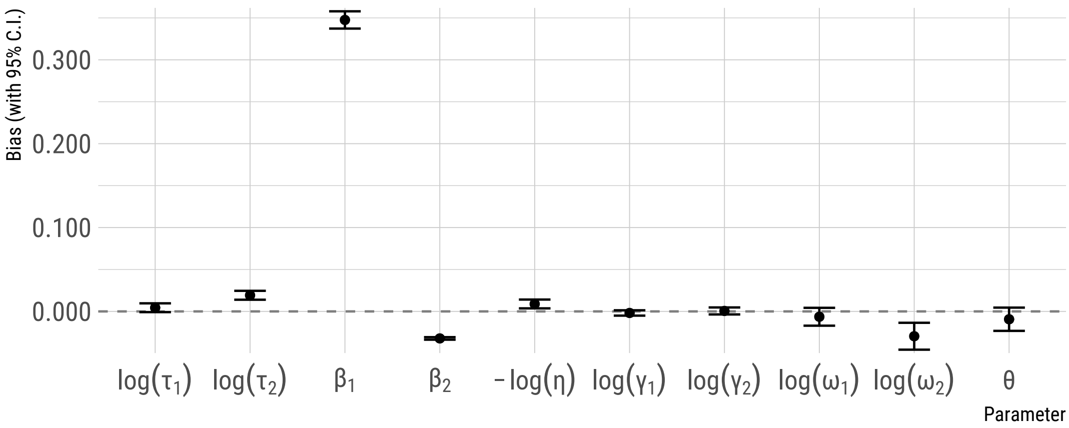

A.2.5 Performance Measures and Number of Replications

The key performance measures of interest are the same ones that were used in the previous simulation study.

To estimate the key performance measure with a given precision, we once again start by running 10 replications of the simulation study. Allowing a Monte Carlo standard error for bias of 0.01, we would require $n_{\text{sim}} = \text{Var}/\text{MCSE}^2 \approx 746$ repetitions of our full simulation study. This calculation was based on assuming $\text{Var} = 0.07464286$, which was taken as the largest value among all empirical and model-based standard errors for $\widehat{\Psi}$ obtained from the above-mentioned 10 replications. To be conservative we rounded up the number of repetitions to the nearest 100, which yielded a total of $n_{\text{sim}} = 800$.

A.2.6 Results

Our model implementation converged for 790 repetitions out of 800 (98.75%); performance measures for the iterations that converged are presented below and tabulated in Table A2.

Figures A6 and A7 describe bias and relative bias for each model parameter. Most parameters could be estimated with no significant absolute bias, or with negligible bias on a relative scale (such as $\log(\tau_2)$, $-\log(\eta)$, and $\log(\omega_2)$). We could however observe significant bias for the parameters of the screening sensitivity model, with overestimation of the intercept β_1 and underestimation of the coefficient for size β_2: this is in agreement with previous work, see e.g. Gasparini and Humphreys (2022), and not surprising given the small number of tumours of very small size at detection. Despite this significant bias, the fitted sensitivity lines did not differ too much from the truth, as illustrated in Figure A8.

Our approach for the estimation of model standard errors was deemed adequate, as the model-based standard errors closely matched empirical standard errors (Figure A9); consequently, relative

Figure A7: Relative bias with inter-quartile intervals, for parameters on the original scale. Simulation study for the model in a screened population.

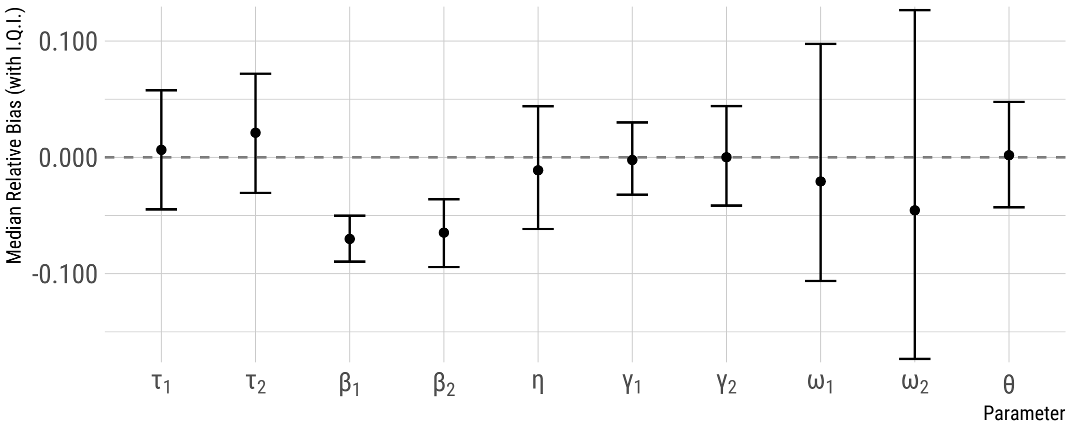

Figure A8: Iteration-specific fitted screening sensitivity functions (in grey) compared to true, data-generating screening sensitivity function (black, dotted line). Simulation study for the model in a screened population.

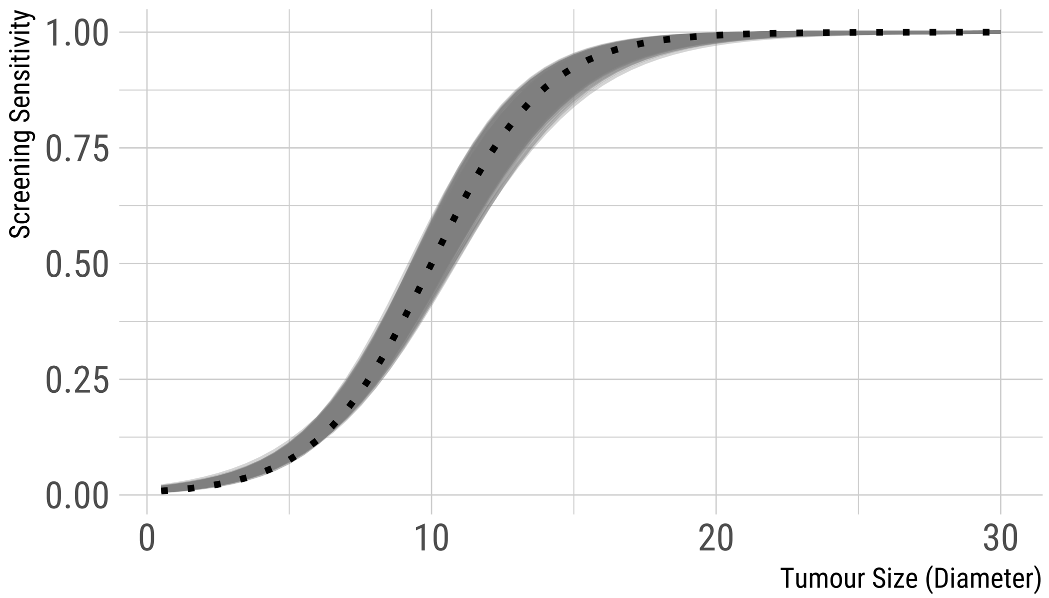

Figure A9: Empirical standard errors and model-based standard errors with 95% confidence intervals based on the Monte Carlo standard errors, simulation study for the model in a screened population.

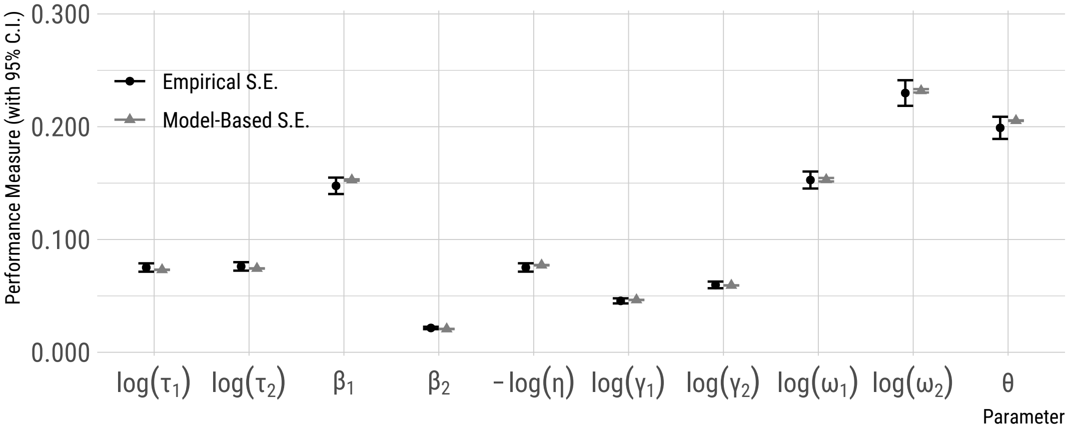

percentage errors in model standard errors were negligible (Figure A10).

Finally, coverage probabilities were optimal for all model parameters except for those with the largest absolute and relative biases, namely the coefficients of the screening sensitivity function β_1 and β_2, for which coverage probability was low at 37.8% and 63.5%, respectively.

Figure A10: Relative percentage error in standard error with 95% confidence intervals based on the Monte Carlo standard errors, simulation study for the model in a screened population.

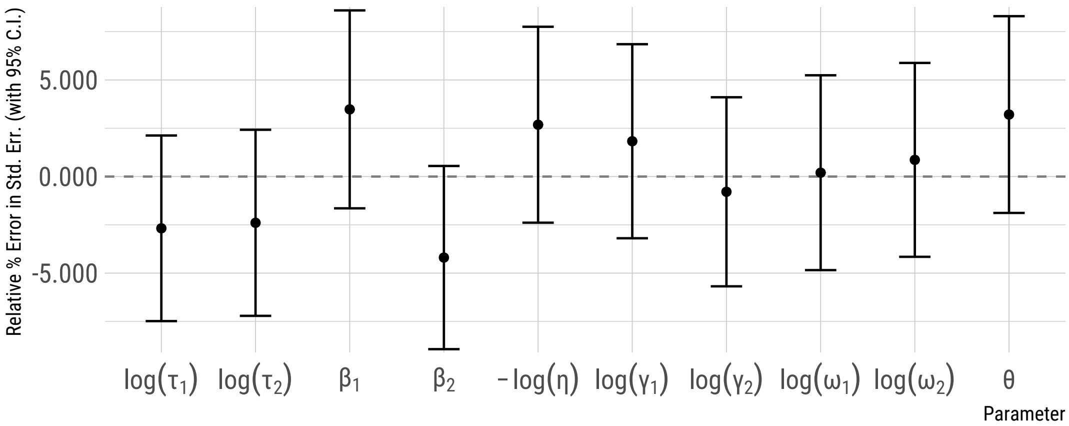

Figure A11: Coverage probability with 95% confidence intervals based on the Monte Carlo standard errors, simulation study for the model in a screened population.

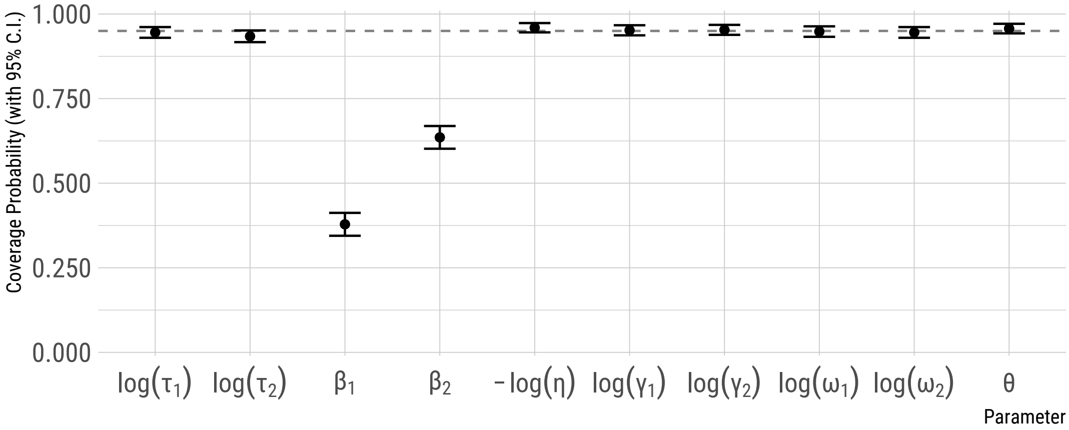

Table A2: Summary statistics for the simulation study to evaluate the estimation procedure for the model in a screened population. Values are point estimates with Monte Carlo standard errors in parentheses.

Parameter	Bias	Emp. S.E.	Model S.E.	Relative Error	Coverage
β_1	0.348 (0.005)	0.148 (0.004)	0.153 (0.000)	3.477 (2.614)	0.378 (0.017)
β_2	-0.032 (0.001)	0.022 (0.001)	0.021 (0.000)	-4.195 (2.420)	0.635 (0.017)
$-\log(\eta)$	0.009 (0.003)	0.075 (0.002)	0.077 (0.000)	2.682 (2.588)	0.959 (0.007)
$\log(\gamma_1)$	-0.002 (0.002)	0.046 (0.001)	0.046 (0.000)	1.828 (2.563)	0.952 (0.008)
$\log(\gamma_2)$	0.001 (0.002)	0.060 (0.002)	0.059 (0.000)	-0.790 (2.497)	0.953 (0.008)
$\log(\omega_1)$	-0.006 (0.005)	0.153 (0.004)	0.153 (0.001)	0.199 (2.573)	0.948 (0.008)
$\log(\omega_2)$	-0.030 (0.008)	0.230 (0.006)	0.232 (0.001)	0.863 (2.560)	0.946 (0.008)
$\log(\tau_1)$	0.004 (0.003)	0.075 (0.002)	0.073 (0.000)	-2.680 (2.451)	0.946 (0.008)
$\log(\tau_2)$	0.019 (0.003)	0.076 (0.002)	0.074 (0.000)	-2.395 (2.458)	0.934 (0.009)
θ	-0.009 (0.007)	0.199 (0.005)	0.205 (0.000)	3.209 (2.598)	0.957 (0.007)

B Additional Results; Analysis of CAHRES Data

Figure B1

Kaplan-Meier curve for the time to detection/diagnosis of distant metastases for patients without detected distant metastasis at diagnosis of the primary tumour (n = 1580).

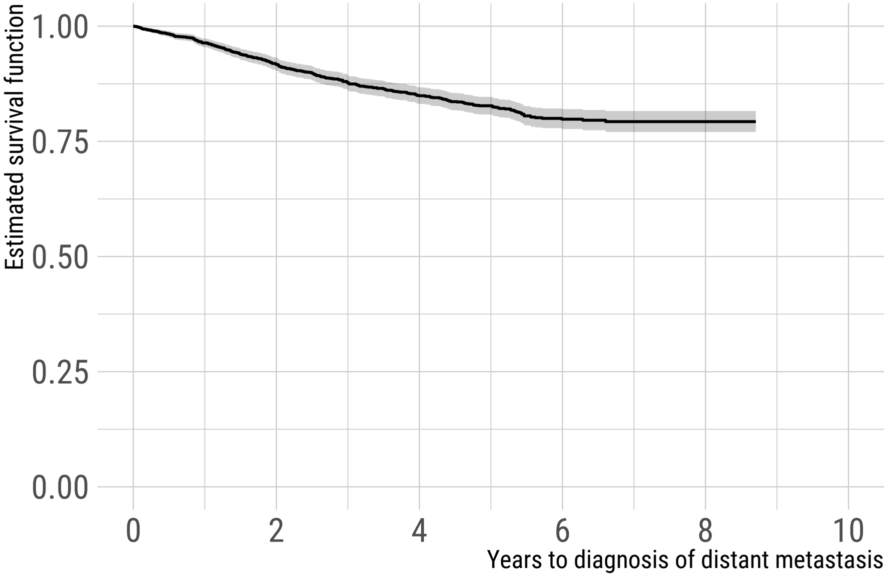

Overall

	0	2	4	6	8	10
At risk	1580	1404	1210	449	30	0
Censored	0	47	140	845	1262	1292
Events	0	129	230	286	288	288

Figure B2

Marginal survival curves (in black) for time to diagnosis of distant metastasis for subjects with zero, one, and two affected lymph nodes at diagnosis. Curves are shown for all models compared in the analysis of the CAHRES data, assuming a Frank, Clayton, AMH, or product copula, and averaged over the observed covariates' distribution in each subgroup. Standard errors are computed using the numerical delta method as implemented in the `predictnl` function from the `rstpm2` package (Liu et al., 2018). Kaplan-Meier curves (in grey) for relevant subgroups of the study population are also included for comparison.

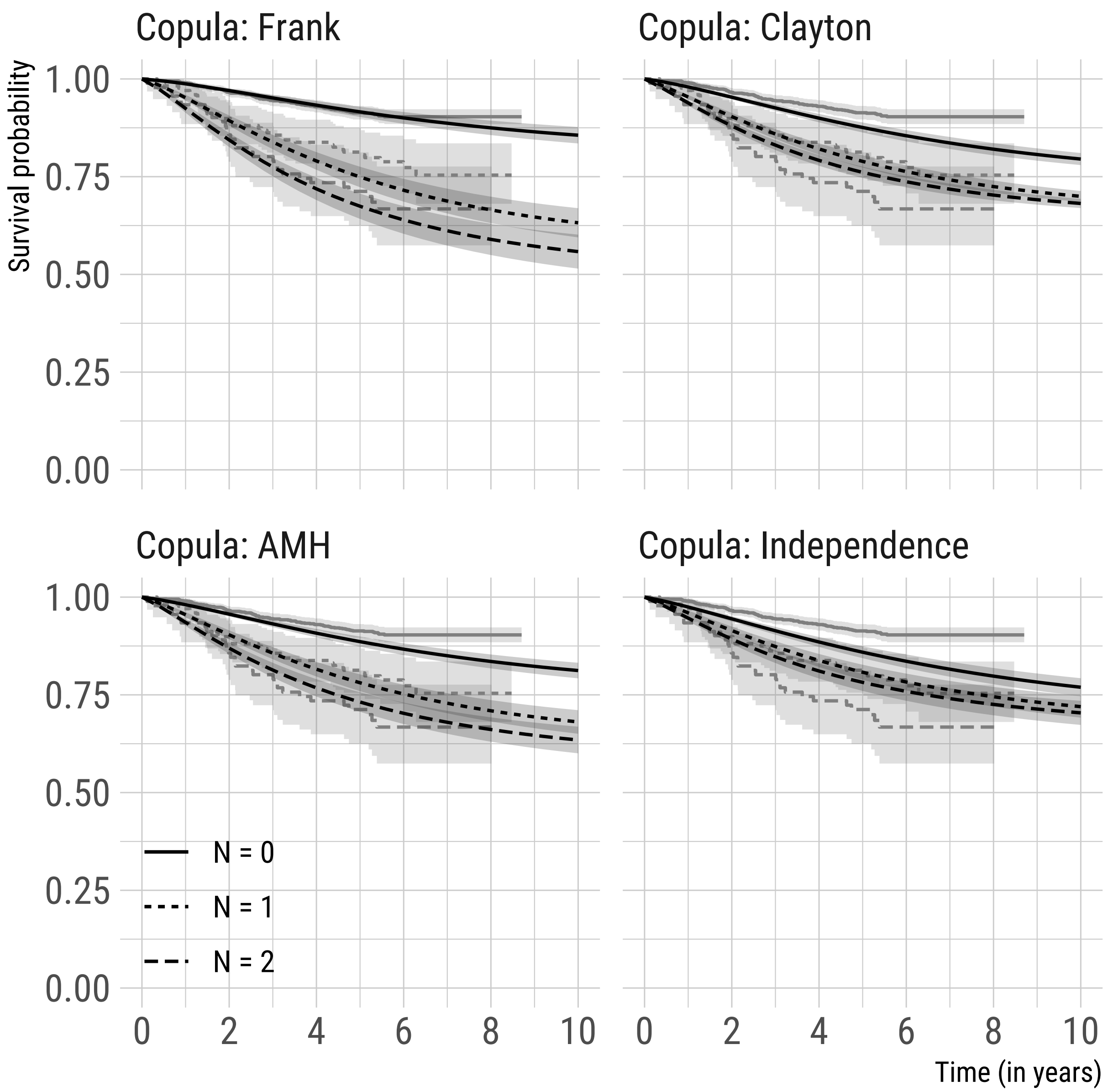

Table B1

Estimated median tumour doubling times according to all models with different copula formulations, application to CAHRES data. Standard errors are computed using the numerical delta method as implemented in the `predictnl` function from the `rstpm2` package (Liu et al., 2018).

Copula	Estimate	Std. Error	95% C.I.
Frank	215.98	3.72	208.69-223.28
Clayton	215.61	3.58	208.59-222.63
AMH	214.51	3.86	206.96-222.07
Independence	212.90	4.01	205.03-220.76

Table B2

Estimated cured proportions under different copula formulations; application to CAHRES data. Values in each cell are cured proportions with 95% confidence intervals, and are presented overall and by number of affected lymph nodes. Standard errors are computed using the numerical delta method as implemented in the `predictnl` function from the `rstpm2` package (Liu et al., 2018).

	Frank	Clayton	AMH	Independence
Overall	0.697 (0.658-0.736)	0.689 (0.684-0.695)	0.680 (0.640-0.720)	0.667 (0.625-0.708)
LN = 0	0.805 (0.772-0.839)	0.726 (0.721-0.732)	0.744 (0.711-0.777)	0.681 (0.642-0.721)
LN = 1	0.553 (0.500-0.605)	0.629 (0.624-0.635)	0.604 (0.560-0.649)	0.648 (0.605-0.692)
LN = 2	0.479 (0.423-0.535)	0.614 (0.608-0.619)	0.559 (0.511-0.606)	0.639 (0.595-0.683)

Table B3

Estimated survival probabilities for time to distant metastasis according to all models with different copula formulations; application to CAHRES data. Values in each cell are survival probabilities with 95% confidence intervals. Standard errors are computed using the numerical delta method as implemented in the `predictnl` function from the `rstpm2` package (Liu et al., 2018).

Time	N = 0	N = 1	N = 2
**Copula: Frank**			
1	0.987 (0.985-0.990)	0.952 (0.945-0.959)	0.924 (0.914-0.935)
2	0.970 (0.965-0.975)	0.893 (0.880-0.907)	0.844 (0.826-0.862)
5	0.916 (0.903-0.929)	0.749 (0.723-0.775)	0.674 (0.642-0.707)
10	0.856 (0.835-0.877)	0.632 (0.595-0.669)	0.558 (0.515-0.602)
**Copula: Clayton**			
1	0.979 (0.977-0.982)	0.954 (0.949-0.959)	0.939 (0.932-0.945)
2	0.953 (0.948-0.958)	0.904 (0.894-0.913)	0.880 (0.869-0.890)
5	0.876 (0.863-0.889)	0.789 (0.773-0.805)	0.761 (0.747-0.775)
10	0.795 (0.780-0.811)	0.700 (0.686-0.713)	0.681 (0.670-0.693)
**Copula: AMH**			
1	0.981 (0.978-0.983)	0.955 (0.948-0.961)	0.935 (0.927-0.944)
2	0.956 (0.951-0.962)	0.904 (0.892-0.915)	0.869 (0.855-0.884)
5	0.886 (0.873-0.899)	0.781 (0.759-0.802)	0.732 (0.707-0.757)
10	0.812 (0.792-0.832)	0.681 (0.650-0.711)	0.634 (0.600-0.669)
**Copula: Independence**			
1	0.975 (0.971-0.978)	0.960 (0.954-0.965)	0.946 (0.939-0.954)
2	0.944 (0.938-0.951)	0.914 (0.904-0.925)	0.892 (0.880-0.905)
5	0.859 (0.844-0.874)	0.808 (0.789-0.827)	0.782 (0.760-0.804)
10	0.769 (0.746-0.793)	0.720 (0.692-0.748)	0.704 (0.673-0.735)

References

R Core Team. *R: A Language and Environment for Statistical Computing*. R Foundation for Statistical Computing, Vienna, Austria, 2021. URL `https://www.R-project.org/`.

A. Gasparini. `rsimsum`: Summarise results from Monte Carlo simulation studies. *Journal of Open Source Software*, 3(26):739, 2018. doi: 10.21105/joss.00739.

R. H. Byrd, P. Lu, J. Nocedal, and C. Zhu. A limited memory algorithm for bound constrained optimization. *SIAM Journal on Scientific Computing*, 16(5):1190–1208, sep 1995. doi: 10.1137/0916069. URL `https://doi.org/10.1137%2F0916069`.

L. Abrahamsson and K. Humphreys. A statistical model of breast cancer tumour growth with estimation of screening sensitivity as a function of mammographic density. *Statistical Methods in Medical Research*, 25(4):1620–1637, 2016. doi: 10.1177/0962280213492843.

G. Isheden and K. Humphreys. Modelling breast cancer tumour growth for a stable disease population. *Statistical Methods in Medical Research*, 28(3):681–702, 2019. doi: 10.1177/0962280217734583.

G. Isheden, L. Abrahamsson, T. Andersson, K. Czene, and K. Humphreys. Joint models of tumour size and lymph node spread for incident breast cancer cases in the presence of screening. *Statistical Methods in Medical Research*, 28(12):3822–3842, 2019. doi: 10.1177/0962280218819568.

A. Gasparini and K. Humphreys. Estimating latent, dynamic processes of breast cancer tumour growth and distant metastatic spread from mammography screening data. *Statistical Methods in Medical Research*, 2022. doi: 10.1177/09622802211072496.

T. P. Morris, I. R. White, and M. J. Crowther. Using simulation studies to evaluate statistical methods. *Statistics in Medicine*, 38(11):2074–2102, 2019. doi: 10.1002/sim.8086.

C. Forastero, L. I. Zamora, D. Guirado, and A. M. Lallena. A monte carlo tool to simulate breast cancer screening programmes. *Physics in Medicine and Biology*, 55(17):5213–5229, 2010. doi: 10.1088/0031-9155/55/17/021.

X.-R. Liu, Y. Pawitan, and M. Clements. Parametric and penalized generalized survival models. *Statistical Methods in Medical Research*, 27(5):1531–1546, 2018.
